# Supplementary figures and images for: Two-step mixed model approach to analyzing differential alternative RNA splicing
Source: PLoS One. 2020 Oct 9;15(10):e0232646. doi: 10.1371/journal.pone.0232646 (PMC7546511; doi:10.1371/journal.pone.0232646)

Figure S1. The isoform expression profiles for three template genes used for simulation study

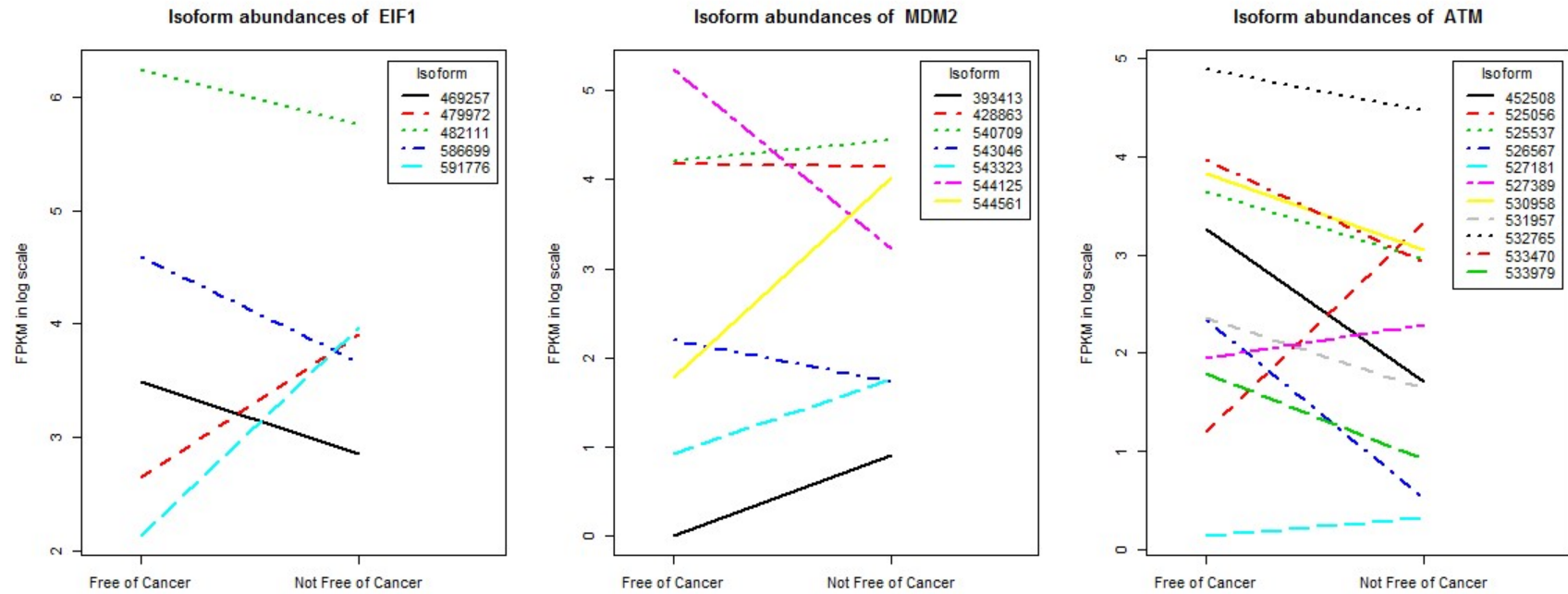

Supplement: S1 Fig — (PDF) [file pone.0232646.s001.pdf]
