# Supplementary material for: Two-step mixed model approach to analyzing differential alternative RNA splicing
Source: PLoS One. 2020 Oct 9;15(10):e0232646. doi: 10.1371/journal.pone.0232646 (PMC7546511; doi:10.1371/journal.pone.0232646)

Figure S5. Evaluation of OFDR and Power through the Simulation with Template Gene MDM2 when t-test is used in the confirmatory stage.

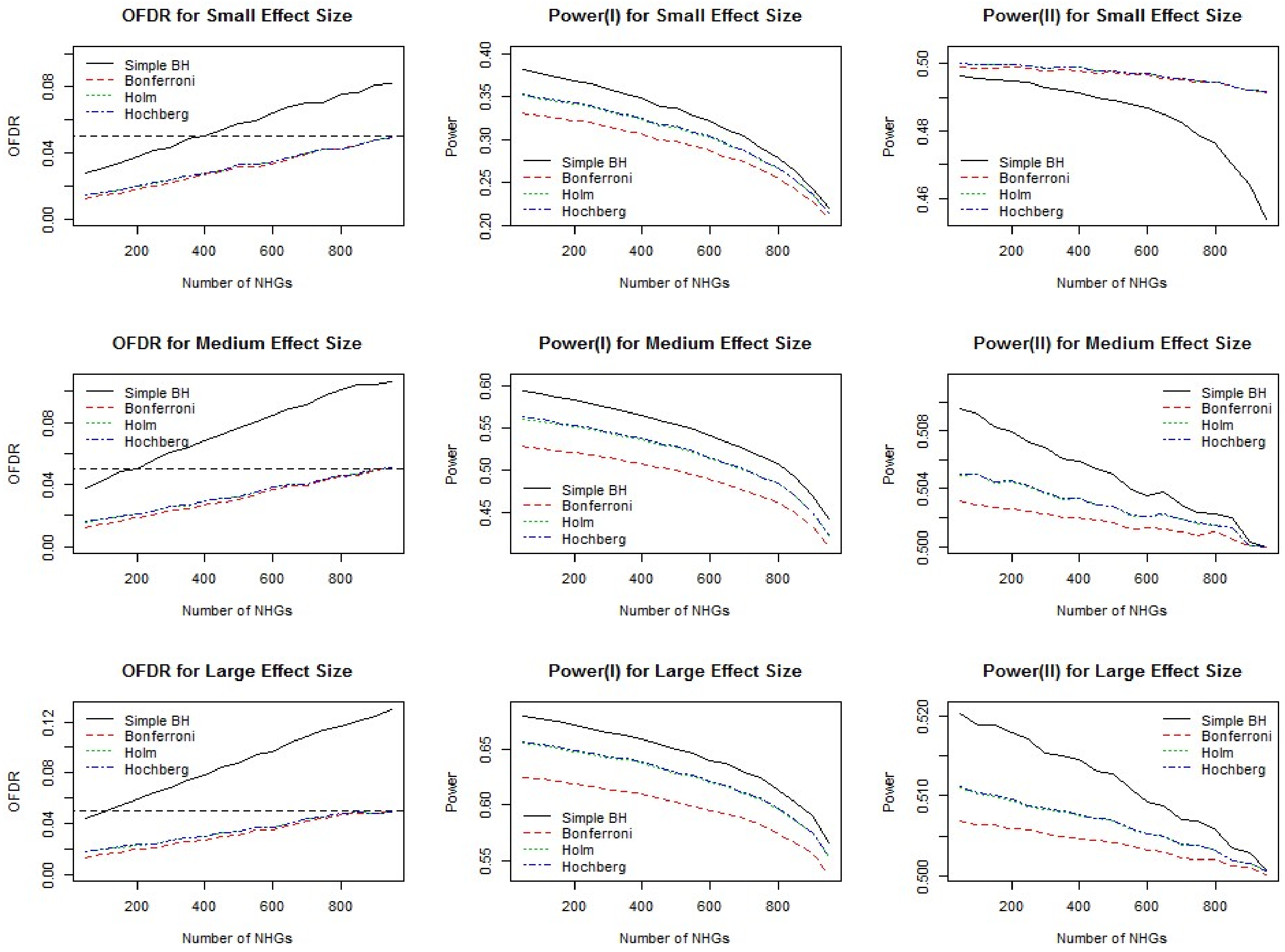

Supplement: S5 Fig — (PDF) [file pone.0232646.s005.pdf]
