# Supplementary material for: Two-step mixed model approach to analyzing differential alternative RNA splicing
Source: PLoS One. 2020 Oct 9;15(10):e0232646. doi: 10.1371/journal.pone.0232646 (PMC7546511; doi:10.1371/journal.pone.0232646)

Figure S7. Simulation with Template Gene EIF1 and Small Sample Size (N=50 in each group) when Wald-test is used in the confirmatory stage.

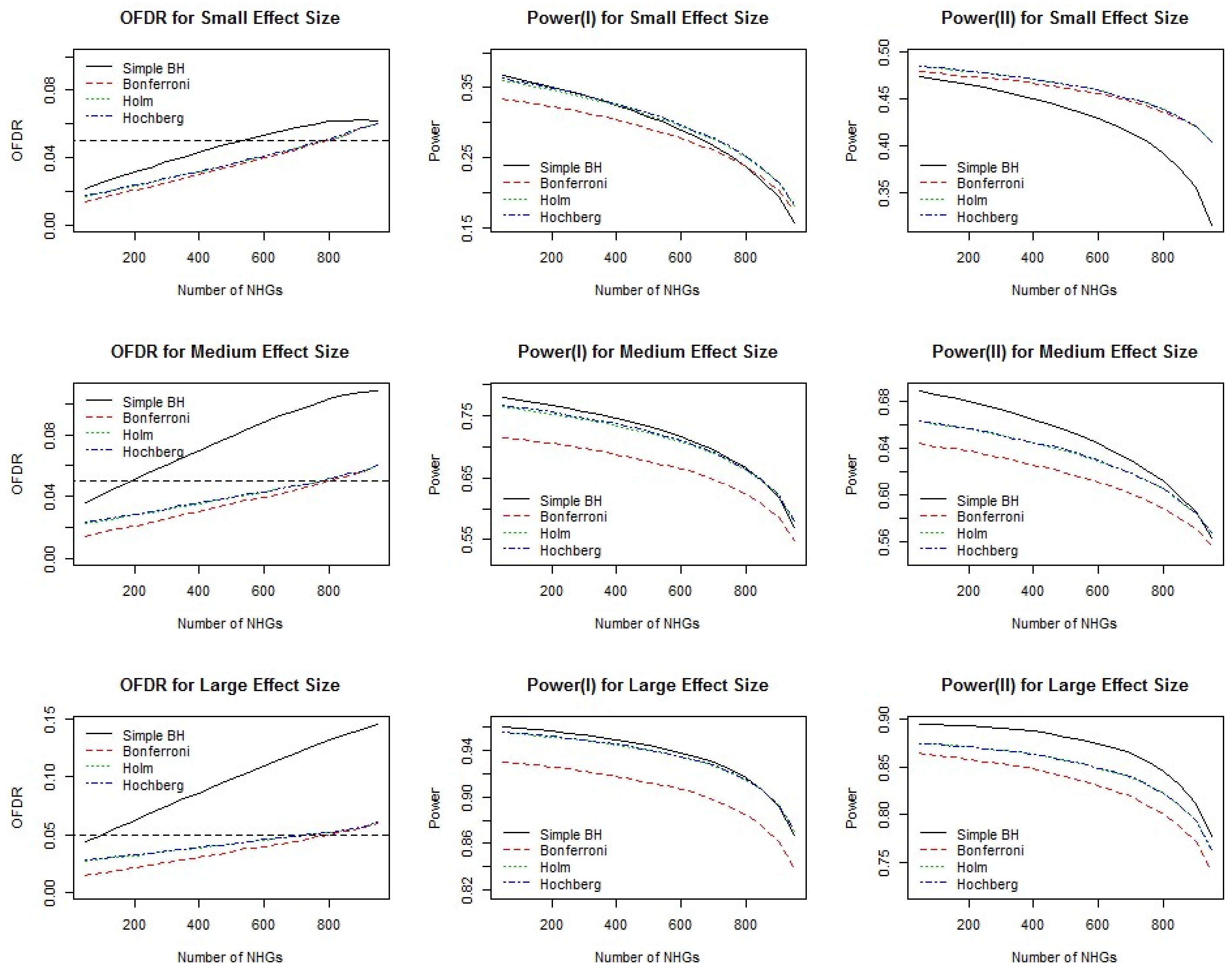

Supplement: S7 Fig — (PDF) [file pone.0232646.s007.pdf]
