# Supplementary material for: Two-step mixed model approach to analyzing differential alternative RNA splicing
Source: PLoS One. 2020 Oct 9;15(10):e0232646. doi: 10.1371/journal.pone.0232646 (PMC7546511; doi:10.1371/journal.pone.0232646)
Supplement: S1 Table — Type 1 screening test identified 50 genes with differentially expressed/spliced isoforms, and type 2 screening test identified 8 genes with differentially spliced genes. Results were obtained from linear mixed model with unstructured covariance structure for ACC Study. (PDF) [file pone.0232646.s010.pdf]

Supplementary Table 1. List of genes that were passed Type 1 and 2 screening tests along with the likelihood ratio test p-values and FDR. Type 1 screening test identified 50 genes with differentially expressed/spliced isoforms, and type 2 screening test identified 8 genes with differentially spliced genes. Results were obtained from linear mixed model with unstructured covariance structure for ACC Study.

Gene Name	Number of Isoforms	Type 1 Screening		Type 2 Screening	
		LRT p-value	FDR	LRT p-value	FDR
gene	nI	p1	fdr1	p2	fdr2
GNB2L1	12	7.00E-10	1.97E-06	1.28E-07	0.00018
NPM1	11	3.72E-08	5.22E-05	1.23E-07	0.00018
TPM1	12	1.90E-06	0.001779		
CD44	12	2.56E-06	0.0018	3.05E-06	0.002857
RSRC2	7	8.84E-06	0.004963		
EPB41L2	9	1.19E-05	0.005582		
MYL6	8	1.42E-05	0.005704		
CSDE1	11	3.12E-05	0.010967		
MATR3	11	7.03E-05	0.013519		
CTNNA1	11	6.11E-05	0.013519		
XPO1	11	7.58E-05	0.013519		
MDM2	7	5.36E-05	0.013519		
SEC31A	9	7.70E-05	0.013519		
NPC1	4	5.68E-05	0.013519		
SDHD	3	5.87E-05	0.013519		
HNRNPA2E	6	8.83E-05	0.014586		
SUPT16H	4	0.000107	0.015837		
VEGFA	2	0.000118	0.016508		
ASXL1	3	0.00013	0.017212		
RPLP2	4	0.000135	0.017212		
HDGF	3	0.000143	0.01751		
BCLAF1	7	7.28E-05	0.013519	3.22E-05	0.022582
SAT1	7	0.000169	0.019721		
SAR1B	3	0.000202	0.02209		
STK39	3	0.000206	0.02209		
TMEM63A	6	0.000101	0.015682	6.81E-05	0.033186
SUPT5H	3	0.000257	0.025759	7.09E-05	0.033186
CCPG1	4	0.000353	0.034196		
FZD6	2	0.000373	0.034267		
NDRG2	10	0.000212	0.02209	0.000134	0.047058
DCN	9	0.000403	0.035402		
CALD1	7	0.000477	0.039959		
CCNL1	8	0.000485	0.039959		
SF3B3	3	0.000498	0.039959		
PDCD4	7	0.000515	0.040205		

Gene Name	Number of Isoforms	Type 1 Screening		Type 2 Screening	
		LRT p-value	FDR	LRT p-value	FDR
PRKAA1	3	0.000378	0.034267	0.000119	0.047058
TFPI	3	0.000543	0.041213		
RALA	2	0.00059	0.041358		
CTTN	6	0.000561	0.041358		
RPLP0	6	0.000621	0.041358		
HECTD1	12	0.000647	0.041358		
RPL36	2	0.000631	0.041358		
ANXA2	9	0.000632	0.041358		
COL27A1	3	0.000648	0.041358		
C3orf17	4	0.000681	0.042522		
LIFR	3	0.00073	0.042694		
TXNIP	5	0.000725	0.042694		
CD46	5	0.000711	0.042694		
RRBP1	3	0.000823	0.047167		
DSC2	2	0.000864	0.048516		
