# Supplementary material for: Two-step mixed model approach to analyzing differential alternative RNA splicing
Source: PLoS One. 2020 Oct 9;15(10):e0232646. doi: 10.1371/journal.pone.0232646 (PMC7546511; doi:10.1371/journal.pone.0232646)
Supplement: S2 Table — Results obtained from the Wald test following the type 1 screening test based on the linear mixed model with unstructured covariance structure for ACC Study. (PDF) [file pone.0232646.s011.pdf]

Supplementary Table 2. List of 18 differentially expressed isoforms between 8 patients who are free of cancer vs. 6 patients in the confirmatory test. Results obtained from the Wald test following the Type 1 screening test based on the linear mixed model with unstructured covariance structure for ACC Study.

| Isoform ID | Gene Name | p value (model-based) | p-value (t test) | Two step Significanc e threshold |
|---|---|---|---|---|
| ENST00000437725 | TFPI | 8.93E-06 | 0.00125 | 0.0003 |
| ENST00000005257 | RALA | 1.64E-05 | 0.0013 | 0.00045 |
| ENST00000521387 | CTNNA1 | 7.10E-08 | 0.00015 | 8.09E-05 |
| ENST00000497139 | VEGFA | 4.52E-06 | 0.00052 | 0.00045 |
| ENST00000263409 | LIFR | 8.80E-07 | 0.00042 | 0.0003 |
| ENST00000356674 | HNRNPA2E | 1.32E-09 | 0.00081 | 0.00015 |
| ENST00000495501 | RRBP1 | 1.68E-05 | 0.00139 | 0.0003 |
| ENST00000511248* | PRKAA1* | 2.92E-07 | 0.00026 | 0.0003 |
| ENST00000280904 | DSC2 | 1.94E-05 | 0.00137 | 0.00045 |
| ENST00000505479 | SEC31A | 6.38E-05 | 0.00708 | 9.89E-05 |
| ENST00000590723 | NPC1 | 1.04E-06 | 0.00025 | 0.00022 |
| ENST00000474311 | C3orf17 | 2.11E-06 | 0.0003 | 0.00022 |
| ENST00000474539 | CCNL1 | 1.25E-08 | 0.00025 | 0.00011 |
| ENST00000522566 | FZD6 | 6.81E-07 | 0.00028 | 0.00045 |
| ENST00000600818* | SUPT5H* | 4.55E-06 | 0.00039 | 0.0003 |
| ENST00000451716 | COL27A1 | 8.94E-07 | 0.00038 | 0.0003 |
| ENST00000355999 | STK39 | 6.96E-07 | 0.00091 | 0.0003 |
| ENST00000310958 | CCPG1 | 2.60E-06 | 0.00034 | 0.00022 |

* The two isoforms that were also called significant by the confirmatory test following Type 2 screening test.
