# Supplementary material for: Two-step mixed model approach to analyzing differential alternative RNA splicing
Source: PLoS One. 2020 Oct 9;15(10):e0232646. doi: 10.1371/journal.pone.0232646 (PMC7546511; doi:10.1371/journal.pone.0232646)
Supplement: S3 Table — Type 1 screening test identified 782 genes with differentially expressed/spliced isoforms, and type 2 screening test identified 857 genes woth differentially spliced genes. Results were obtained from linear mixed model with unequal variance covariance structure for AML Study. (PDF) [file pone.0232646.s012.pdf]

Supplementary Table 3. List of genes that were passed Type 1 and 2 screening tests along with the likelihood ratio test p-values and FDR. Type 1 screening test identified 782 genes with differentially expressed/spliced isoforms, and type 2 screening test identified 857 genes woth differentially spliced genes. Results were obtained from linear mixed model with unequal variance covariance structure for AML Study.

Gene Name	Number of Isoforms	Type 1 Screening		Type 2 Screening	
		LRT p-value	FDR	LRT p-value	FDR
TARDBP	8	4.14E-14	3.24E-10	1.88E-14	1.47E-10
SP100	14	1.74E-12	6.82E-09	2.69E-11	7.02E-08
MYCBP2	7	3.37E-11	8.80E-08	1.42E-11	5.54E-08
NCK1	7	4.54E-10	8.88E-07	1.75E-10	3.43E-07
SPAG9	11	6.66E-10	1.04E-06	4.67E-10	6.09E-07
UBXN1	13	9.88E-10	1.29E-06	3.98E-10	6.09E-07
GLYR1	9	1.16E-08	1.14E-05	5.10E-09	5.71E-06
EIF4G2	20	9.66E-09	1.08E-05	7.00E-09	6.85E-06
EEF1B2	8	4.08E-08	3.19E-05	1.90E-08	1.65E-05
KANSL2	9	8.97E-08	5.40E-05	3.39E-08	2.65E-05
FAM214A	7	1.03E-07	5.73E-05	4.10E-08	2.91E-05
WDR6	8	1.43E-07	7.00E-05	5.22E-08	3.40E-05
RPL15	7	1.78E-07	8.19E-05	6.40E-08	3.85E-05
DMTF1	11	1.12E-07	5.86E-05	1.43E-07	7.61E-05
CCNL1	17	7.50E-08	4.89E-05	1.92E-07	9.41E-05
ZRANB2	4	3.90E-08	3.19E-05	4.19E-07	0.000153
ANKHD1	13	2.94E-07	0.000121	1.46E-07	7.61E-05
TPP2	7	2.75E-07	0.000119	4.31E-07	0.000153
KTN1	11	5.88E-07	0.00017	2.53E-07	0.000117
RB1CC1	6	4.33E-07	0.000147	3.86E-07	0.000153
TBC1D23	7	5.39E-07	0.000162	4.17E-07	0.000153
TTC7A	11	4.31E-07	0.000147	6.41E-07	0.000209
MST4	5	9.93E-07	0.000243	3.17E-07	0.000138
OXA1L	7	4.82E-07	0.000151	8.51E-07	0.00025
PCM1	14	8.54E-07	0.00023	7.07E-07	0.000221
VDAC2	8	1.32E-06	0.000296	6.06E-07	0.000206
ATP2C1	10	9.20E-07	0.000234	1.04E-06	0.000281
U2AF2	7	1.40E-06	0.000296	9.13E-07	0.000255
SOCS2	6	4.87E-08	3.47E-05	2.89E-06	0.000518
PTPN22	4	1.43E-06	0.000296		
PAPOLA	13	1.84E-06	0.000342	8.64E-07	0.00025
DICER1	7	1.50E-06	0.000301	1.26E-06	0.000318
CCPG1	7	1.67E-06	0.000323	1.14E-06	0.000297
TXNRD1	9	6.70E-07	0.000187	2.54E-06	0.000474
ROCK1	5	4.46E-07	0.000147	3.10E-06	0.000518
LEMD2	9	1.44E-06	0.000296	1.60E-06	0.00038
FUBP1	9	1.43E-06	0.000296	2.22E-06	0.000445

Gene Name	Number of Isoforms	Type 1 Screening		Type 2 Screening	
		LRT p-value	FDR	LRT p-value	FDR
HMGN1	12	2.08E-06	0.000379	1.73E-06	0.000392
TRA2B	13	2.85E-06	0.000448	1.34E-06	0.000328
CHMP1A	6	2.38E-06	0.000405	1.77E-06	0.000392
EIF4B	8	2.27E-06	0.000395	2.33E-06	0.000456
C1orf63	15	3.13E-06	0.00047	1.80E-06	0.000392
TAF1D	17	2.86E-06	0.000448	2.12E-06	0.000437
PABPC1	18	2.92E-06	0.000448		
CSAD	4	3.27E-06	0.000483	2.46E-06	0.00047
ARMC8	15	3.81E-06	0.000553	1.96E-06	0.000414
SRSF5	15	4.70E-06	0.000656	3.11E-06	0.000518
ATP2B1	11	6.48E-06	0.000831	2.95E-06	0.000518
43896	9	7.10E-06	0.000875	2.99E-06	0.000518
BCLAF1	16	2.57E-06	0.000427	8.27E-06	0.000985
RBM26	5	2.18E-06	0.000387	9.41E-06	0.001037
OSBPL9	12	7.31E-06	0.000875	3.41E-06	0.000556
NAP1L4	11	7.88E-06	0.000881	3.74E-06	0.000595
HNRNPK	11	7.81E-06	0.000881	3.80E-06	0.000595
COMMD3	10	9.26E-07	0.000234	1.25E-05	0.001256
PPP1CC	9	6.12E-06	0.000822	5.16E-06	0.000722
TSPAN32	7	7.17E-06	0.000875	5.17E-06	0.000722
NPR3	4	4.01E-06	0.000571	9.17E-06	0.001033
ELF2	8	1.04E-05	0.00106	4.26E-06	0.000654
BZW2	11	1.69E-06	0.000323	1.66E-05	0.001444
TSPAN7	3	8.12E-06	0.000895	7.07E-06	0.000907
IBTK	6	1.17E-05	0.001143	4.50E-06	0.000678
44076	12	1.09E-05	0.001094	5.30E-06	0.000728
PRPF40A	9	6.20E-06	0.000822	8.75E-06	0.001022
43897	4	1.14E-05	0.001133	4.93E-06	0.000722
ARID4A	8	1.27E-05	0.001199	5.88E-06	0.000767
HERC4	6	1.38E-05	0.001272	5.47E-06	0.000738
FAM91A1	7	1.49E-05	0.001337	5.80E-06	0.000767
SLC25A5	4	1.67E-05	0.001388	5.14E-06	0.000722
LONRF1	3	9.61E-06	0.001029	1.05E-05	0.001095
LBR	6	1.20E-05	0.001145	8.30E-06	0.000985
TBC1D15	9	1.20E-05	0.001145	9.24E-06	0.001033
DHX15	8	7.60E-06	0.000881	1.32E-05	0.00131
CLK1	13	1.30E-05	0.00121	9.72E-06	0.001057
CD46	12	2.81E-06	0.000448	2.54E-05	0.001843
U2SURP	11	1.65E-05	0.001388	8.25E-06	0.000985
FBXO11	8	1.78E-05	0.001453	7.84E-06	0.000974
HSPA9	9	1.63E-05	0.001388	9.91E-06	0.001063
HERC1	7	1.98E-05	0.001536	7.79E-06	0.000974
IQCB1	3	8.27E-06	0.000899	2.13E-05	0.001671
SEH1L	6	7.10E-06	0.000875	2.22E-05	0.001723

Gene Name	Number of Isoforms	Type 1 Screening		Type 2 Screening	
		LRT p-value	FDR	LRT p-value	FDR
CTNNB1	9	1.63E-05	0.001388	1.41E-05	0.001311
GPR114	4	1.61E-05	0.001388	1.44E-05	0.001328
STX16	7	2.30E-05	0.001699	9.12E-06	0.001033
DEK	6	1.66E-05	0.001388		
FAM120AOS	8	2.30E-05	0.001699	1.06E-05	0.001095
ZFYVE27	7	1.49E-05	0.001337	1.73E-05	0.001458
ZDHHC6	3	7.88E-06	0.000881	2.83E-05	0.001932
CLK3	18			1.60E-05	0.001422
SLTM	14	1.98E-05	0.001536	1.36E-05	0.001311
ZBTB43	4	2.07E-05	0.001569	1.39E-05	0.001311
BPTF	8	1.63E-05	0.001388	1.83E-05	0.001511
FNBP4	10	2.06E-05	0.001569	1.49E-05	0.001361
PHTF2	4	9.88E-06	0.001044	2.86E-05	0.001932
POLR2B	6	1.83E-05	0.001476	1.91E-05	0.001525
RTN4	10	2.09E-05	0.001569	1.68E-05	0.001444
SRSF11	16	1.78E-05	0.001453	2.14E-05	0.001671
MED14	3	2.94E-05	0.002041	1.14E-05	0.001156
LMNA	13	2.73E-05	0.001945	1.39E-05	0.001311
CDKN1C	4	3.31E-05	0.002213	1.06E-05	0.001095
RUNX2	6	3.29E-05	0.002213	1.40E-05	0.001311
PSMA4	10	2.87E-05	0.002024	1.86E-05	0.001517
DYNC1I2	8	3.07E-05	0.002088	1.73E-05	0.001458
GCA	3	5.30E-06	0.000728	4.82E-05	0.002926
TMEM87A	5	3.44E-05	0.002258	1.67E-05	0.001444
CLK4	8	1.04E-05	0.00106	4.38E-05	0.002697
RIT1	5	3.69E-05	0.002364	1.56E-05	0.001401
MRPS35	5	6.31E-06	0.000823	4.98E-05	0.00296
RPS27A	9	3.53E-05	0.002298	1.83E-05	0.001511
DMXL2	7	7.38E-06	0.000875	5.42E-05	0.003072
ATP11B	6	4.21E-05	0.002633	1.90E-05	0.001525
WDR48	9	3.73E-05	0.002375	2.84E-05	0.001932
THOC2	8	1.04E-05	0.00106	6.49E-05	0.003434
SMEK1	10	4.61E-05	0.002794	2.40E-05	0.001775
CEP85	4	4.97E-05	0.002899	2.30E-05	0.001741
ANKRA2	3	1.85E-05	0.001476	5.77E-05	0.003182
FBXO3	4	5.42E-05	0.003045	2.31E-05	0.001741
PITPNB	3	5.09E-05	0.002899	2.66E-05	0.001895
INSIG1	6	5.11E-05	0.002899	3.06E-05	0.002045
RICTOR	5	6.06E-05	0.003269	2.31E-05	0.001741
SATB1	7	2.36E-05	0.001722	6.48E-05	0.003434
ZMYM2	7	6.06E-05	0.003269	2.74E-05	0.001932
ASUN	3	4.25E-05	0.002633	4.12E-05	0.002602
UBE2S	2	6.38E-05	0.003325	2.80E-05	0.001932
PER1	16	5.61E-05	0.003091	3.40E-05	0.002244

Gene Name	Number of Isoforms	Type 1 Screening		Type 2 Screening	
		LRT p-value	FDR	LRT p-value	FDR
STAG2	5	7.16E-05	0.003556	2.60E-05	0.001864
NPM3	3	3.59E-05	0.00232	5.52E-05	0.003111
SPNS3	4	3.40E-05	0.002254	5.75E-05	0.003182
SLC38A2	11	3.94E-05	0.002486	5.29E-05	0.003044
KNTC1	7	4.51E-07	0.000147	0.000138	0.005488
SRSF2	8	2.44E-05	0.001767	8.29E-05	0.00395
CSDE1	15	6.61E-05	0.003425	3.52E-05	0.002293
RPL17-C18orf32	3	8.34E-05	0.003953	2.38E-05	0.001775
NIN	12	6.86E-05	0.003509	3.41E-05	0.002244
CHD3	7	4.60E-05	0.002794	5.04E-05	0.002968
PNN	4	8.15E-05	0.003937	2.83E-05	0.001932
AQR	4	4.28E-05	0.002633	5.93E-05	0.003247
EIF4G1	23	5.78E-05	0.003162	4.51E-05	0.002759
TCEA1	3	9.43E-05	0.004237	2.54E-05	0.001843
NUCB2	14	1.88E-05	0.001483	0.000112	0.004761
RIMKLB	6	5.61E-05	0.003091	6.09E-05	0.003311
PPWD1	7	4.70E-05	0.002809	7.13E-05	0.003624
TMX3	2	6.29E-05	0.003325		
CYLD	11	8.79E-05	0.004087	4.35E-05	0.002697
RNF138	5	0.000102	0.004489	3.62E-05	0.002343
MED17	10	6.80E-05	0.003499	6.92E-05	0.003587
UBE3A	3	4.81E-05	0.002853	9.24E-05	0.004253
SAFB	13	8.83E-05	0.004087	5.33E-05	0.003044
SENP7	5	6.27E-05	0.003325	8.38E-05	0.00395
TCP1	14	4.69E-05	0.002809	0.000111	0.004752
STAT1	6	9.10E-05	0.004139	6.96E-05	0.003587
AHCTF1	8	0.000112	0.004828	4.92E-05	0.00296
BOD1L1	5	5.45E-05	0.003045	0.000115	0.004783
RPS8	8	0.000115	0.004909	5.14E-05	0.003002
CNOT7	5	8.93E-05	0.004094	8.34E-05	0.00395
PLEKHM3	4	0.000132	0.005432	4.35E-05	0.002697
RBM25	7	0.000124	0.005202	5.28E-05	0.003044
POLR2J2	3	0.000144	0.005828	3.89E-05	0.002496
PKN2	5	0.000131	0.005415	4.99E-05	0.00296
CHD8	6	5.04E-05	0.002899	0.00014	0.005504
TMEM128	3	0.000148	0.005933	4.03E-05	0.002565
DPM1	4	0.000134	0.005488	5.70E-05	0.003182
NEMF	11	8.28E-05	0.003951	0.000111	0.004752
LRMP	9	8.81E-05	0.004087	0.000111	0.004752
MTSS1	7	0.000115	0.004909	8.05E-05	0.00395
AKAP2	2	0.0001	0.004467		
SCAF11	10	9.24E-05	0.004179	0.000113	0.004783
FNBP1	6	7.66E-05	0.003768	0.000131	0.005288
EAPP	2	6.33E-05	0.003325	0.000155	0.005872

Gene Name	Number of Isoforms	Type 1 Screening LRT p-value	Type 1 Screening FDR	Type 2 Screening LRT p-value	Type 2 Screening FDR
NDUFB5	8	0.000144	0.005828	6.43E-05	0.003434
TRIP12	12	0.000122	0.00512	9.29E-05	0.004253
MGEA5	11	0.000105	0.004603	0.000114	0.004783
ADH5	5	0.000158	0.006193	6.20E-05	0.003344
BLCAP	2	8.11E-05	0.003937	0.000145	0.005611
RWDD4	3	6.98E-05	0.003525	0.000169	0.00616
EIF4G3	9	0.000131	0.005415	0.000104	0.004582
SRSF7	8	8.22E-05	0.003943	0.000171	0.006173
TOP2B	6	1.36E-06	0.000296	0.000366	0.009871
DCK	3	0.000179	0.00677	7.01E-05	0.003587
PDS5B	4	0.000185	0.006849	7.00E-05	0.003587
ITGA4	8	0.000162	0.006346	9.11E-05	0.004217
DUT	6	0.000169	0.006525	8.75E-05	0.004099
TNKS1BP1	3	0.000185	0.006849	7.66E-05	0.00383
UBQLN1	5	0.000191	0.00694	7.54E-05	0.003809
KDM3A	6	0.000187	0.006885	8.24E-05	0.00395
CLIP1	12	0.000167	0.006518	9.55E-05	0.004319
KDM6A	7	4.87E-05	0.002862	0.000251	0.00802
NKTR	8	0.000171	0.006528	9.88E-05	0.004443
MYNN	4	0.000212	0.007385	6.97E-05	0.003587
ADAM8	6			0.000139	0.005496
ANKRD12	5	0.000204	0.007281	7.68E-05	0.00383
TGIF1	9	0.000103	0.004507	0.000201	0.006771
GIT2	13	0.000155	0.006164	0.000126	0.005154
CCAR1	10	0.000114	0.004905	0.000183	0.006426
RAB1A	4	0.00022	0.007505	8.35E-05	0.00395
YTHDF2	5	0.000223	0.007505	8.25E-05	0.00395
JMJD6	6	0.000189	0.006936	0.000106	0.004644
CCT5	9	0.000171	0.006528	0.000123	0.005088
PCYT1A	3	8.95E-05	0.004094	0.000231	0.007526
TARS	10	0.000203	0.007281	0.000102	0.004545
ATP8B4	9	0.00022	0.007505	0.000115	0.004783
LMF2	5	0.000252	0.008133	9.46E-05	0.004305
ZNF384	8	0.00017	0.006528	0.00017	0.00616
DCAF16	3	0.000289	0.008833	7.95E-05	0.003936
FNDC3A	5	0.000182	0.006821	0.000161	0.005953
ZNF92	3	5.09E-05	0.002899	0.000372	0.00991
MKNK1	8	0.000193	0.006973	0.000159	0.00591
MSL2	5	0.000257	0.008214	0.000107	0.004676
SAT2	8	0.00023	0.007685	0.000128	0.005235
PRPF4B	8	0.000211	0.007385	0.000146	0.005611
VCL	3	0.00029	0.008833	9.08E-05	0.004217
ANTXR2	5	0.000235	0.007764	0.000131	0.005288
FPGS	10	0.000198	0.007154	0.000159	0.00591

Gene Name	Number of Isoforms	Type 1 Screening		Type 2 Screening	
		LRT p-value	FDR	LRT p-value	FDR
ZNF326	5	0.00027	0.008526	0.000102	0.004545
SIRT6	3	0.000177	0.006706		
DSN1	3	0.000145	0.005828	0.000238	0.007673
CENPC	5	0.000293	0.008833	0.00011	0.004752
UFM1	4	0.000218	0.007505	0.000177	0.006314
LPCAT1	2	2.73E-05	0.001945	0.000507	0.011976
HMGN2	8	6.92E-05	0.003513	0.000409	0.010428
TMED5	5	0.000296	0.008833	0.000124	0.005128
BUB3	5	0.000222	0.007505	0.000188	0.006476
USP10	8	0.000279	0.008702	0.000136	0.005443
PUM1	9	0.000235	0.007764	0.000188	0.006476
USP16	7	0.000289	0.008833	0.000136	0.005443
RAD23B	3	0.000212	0.007385		
DOCK8	10	0.000254	0.008174	0.000196	0.006646
VEZT	9	0.000295	0.008833	0.00017	0.00616
AZI2	5	0.000221	0.007505		
SMARCA5	2	0.000417	0.011123	8.12E-05	0.00395
CDC14A	5	0.000244	0.007939	0.000214	0.007158
IARS	5	0.000276	0.008625	0.000187	0.006476
YBX3	13	0.000283	0.008756	0.000195	0.006646
EDEM3	2	0.000232	0.00771		
RGS10	2	0.000237	0.007784		
TBCD	4	0.000338	0.009729	0.000158	0.005907
GOLGA4	5	0.000337	0.009729	0.000157	0.005907
SBF2	6	0.000354	0.01	0.000149	0.005698
RNF149	6	0.000222	0.007505	0.000264	0.008307
KLHDC2	7	0.000244	0.007939		
KIAA1109	7	0.00027	0.008526	0.000227	0.00744
HNRNPM	2	7.18E-05	0.003556	0.000543	0.012464
TBP	3	0.000378	0.010587	0.000145	0.005611
PRRC2C	8	0.000371	0.010442	0.000173	0.006212
DDX3X	5	0.000399	0.011029	0.000151	0.00574
PLIN3	4	0.000429	0.011305	0.000146	0.005611
UBE2E1	6	0.000404	0.011036	0.00017	0.00616
MSL3	6	0.000206	0.007281	0.000386	0.0101
LMBR1	5	0.000408	0.011048	0.000181	0.006405
NOP58	5	0.000249	0.008096	0.000338	0.009526
DIS3	4	0.000226	0.007599	0.000379	0.010031
TOPBP1	3	0.000296	0.008833		
ACTN1	7	7.07E-05	0.003546	0.000645	0.014158
MOB1A	3	0.000496	0.012158	0.000144	0.005611
FBXL5	7	0.000301	0.008944	0.000301	0.008934
DLD	7	0.000433	0.011339	0.000192	0.006562
MAT2B	6	0.000343	0.009782	0.000265	0.008307

Gene Name	Number of Isoforms	Type 1 Screening		Type 2 Screening	
		LRT p-value	FDR	LRT p-value	FDR
BAD	2	0.000408	0.011048	0.00021	0.007067
NAA16	4	0.000465	0.011707	0.000184	0.006441
BRD1	5	0.000466	0.011707	0.00019	0.006529
RCOR3	9	0.000402	0.011036	0.000223	0.007333
HDAC1	7	0.000354	0.01	0.000286	0.00863
GNG2	6	0.000379	0.010594	0.000265	0.008307
LGALS8	8	0.000316	0.009299	0.000347	0.009618
CORO1C	6	0.00034	0.009729	0.000318	0.009278
BRWD1	5	0.000439	0.011403	0.000236	0.007618
KANSL1	12	0.000445	0.011483	0.000235	0.007618
TLK2	7	0.000473	0.011782	0.000222	0.007327
SNX8	3	0.00055	0.012996	0.000182	0.006426
GAK	12	0.000432	0.011339	0.000256	0.008137
MTR	5	0.00051	0.012416	0.00022	0.007289
STARD3	12	0.000416	0.011123	0.000283	0.00863
USP15	11	0.000304	0.009009	0.000427	0.010792
PPP6R3	12	0.000446	0.011483	0.000272	0.008445
C18orf21	4	0.000257	0.008214	0.000484	0.011728
CANX	9	0.000294	0.008833	0.000455	0.011223
PHF21A	7	2.95E-05	0.002041	0.000968	0.018105
NR2C1	7	0.000208	0.007331	0.000568	0.012888
ATP6AP2	4	0.00039	0.010856	0.000328	0.009432
CUL3	8	0.000467	0.011707	0.000283	0.00863
ZNF195	6	0.000397	0.011021	0.000321	0.009343
NPEPPS	9	0.000495	0.012158	0.000262	0.008307
AAMP	8	0.000463	0.011707	0.000296	0.008835
KDM4C	8	0.000408	0.011048	0.000378	0.010029
SCP2	6	0.000205	0.007281	0.000635	0.013995
HPGDS	2	0.000632	0.01402	0.00022	0.007289
PPP1R12A	8	0.000439	0.011403	0.000371	0.00991
PTCD3	6	0.000418	0.011123	0.000396	0.010242
ZFC3H1	6	0.000318	0.009323	0.000514	0.012045
CDCA7	6	0.000456	0.011648	0.000363	0.009857
TANK	6	0.000273	0.008588	0.000587	0.013207
EDC4	10	0.000556	0.013088	0.000291	0.008723
KIAA1033	3	0.000182	0.006821	0.000731	0.015305
RABGGTB	8	0.000526	0.012664	0.000336	0.009489
ATP2B4	6	0.000536	0.012812	0.000333	0.009489
ZZEF1	6	0.000638	0.01402	0.000269	0.008397
C7orf55-LUC7L2	8	0.000568	0.013264	0.000317	0.009278
MKRN1	7	0.000636	0.01402	0.000284	0.00863
RPGR	3	0.000648	0.014207	0.000285	0.00863
NDRG1	9	0.000616	0.013849	0.000308	0.009088
SPPL2A	3	0.000446	0.011483		

Gene Name	Number of Isoforms	Type 1 Screening		Type 2 Screening	
		LRT p-value	FDR	LRT p-value	FDR
CEP95	11	0.000588	0.013489	0.000344	0.009592
MBNL2	3	0.000736	0.01547	0.000239	0.007673
RALGAPA2	2	0.000849	0.016772	0.00018	0.006405
SAT1	9	0.000616	0.013849	0.000326	0.009432
CHD9	9	0.000578	0.013369	0.000382	0.010037
SMARCE1	11	0.00046	0.011691	0.000484	0.011728
KDM3B	7	0.000626	0.014018	0.000327	0.009432
DNM2	14	0.000569	0.013264	0.000392	0.010218
EIF4E3	2	0.00047	0.011754		
WRNIP1	3	0.000156	0.006164	0.000893	0.017472
ARID4B	9	0.000495	0.012158	0.000468	0.011494
THOC1	10	0.000637	0.01402	0.000353	0.009672
ASH1L	4	0.0007	0.015018	0.00029	0.00872
EED	4	0.00028	0.008703	0.000722	0.015151
CCDC59	4	7.91E-05	0.003865	0.001153	0.020017
EZH2	8	0.000652	0.01425	0.000353	0.009672
ZCCHC11	11	0.000627	0.014018	0.000372	0.00991
CCNJ	2	0.000486	0.012031		
OCIAD1	11	0.000659	0.014355	0.000365	0.009871
VCP	7	0.000635	0.01402	0.000394	0.010236
SMAD4	5	0.000729	0.015366	0.0003	0.008917
PPM1B	7	0.000703	0.015018	0.000318	0.009278
SACM1L	4	0.000765	0.015728	0.000278	0.008593
SLC25A3	9	0.000607	0.013848	0.000434	0.010898
SLMAP	6	0.000518	0.012505	0.000531	0.012258
CCDC88A	9	0.000541	0.012872	0.000509	0.011976
MICU2	5	0.000545	0.012922	0.000507	0.011976
GLUL	8	0.000696	0.015018	0.000382	0.010037
TPM2	2	0.000151	0.006019	0.001066	0.019243
DLG1	7	0.000762	0.01572	0.000343	0.009583
VPS54	4	0.000118	0.005001	0.001172	0.020303
PHF19	4	0.000785	0.016009	0.000334	0.009489
ACRBP	3	6.37E-05	0.003325	0.001396	0.02234
SNX13	4	0.00045	0.011545	0.000688	0.014604
PAFAH1B1	8	0.00078	0.015979	0.000395	0.010242
ANKRD10	9	0.000741	0.015486	0.000431	0.010855
GSAP	3	0.000571	0.013264		
DENND1B	4	0.000864	0.016976	0.000348	0.009618
TMEM14B	8	0.000758	0.015682	0.000446	0.011057
YPEL5	8	0.000612	0.013849	0.000575	0.013
KAT6B	6	0.000702	0.015018	0.000494	0.011906
FMR1	8	0.000845	0.01676	0.000401	0.0103
BNIP2	9	0.000535	0.012812	0.000654	0.014296
PICALM	6	0.000717	0.01519	0.000501	0.011952

Gene Name	Number of Isoforms	Type 1 Screening		Type 2 Screening	
		LRT p-value	FDR	LRT p-value	FDR
PNP	5	0.000797	0.016155	0.000446	0.011057
VPS29	9	0.000422	0.011195	0.000804	0.016298
MED13L	8	0.000836	0.016716	0.000438	0.010952
ZADH2	3	0.000969	0.018258	0.000335	0.009489
RPL8	11	0.000705	0.015023	0.000563	0.012813
C9orf72	4	0.00011	0.004759	0.001479	0.023154
SS18	4	0.000965	0.018258	0.000351	0.009664
ABCA1	2	0.000967	0.018258	0.000356	0.0097
MRE11A	2	0.000638	0.01402		
MAX	8	0.000615	0.013849	0.000667	0.01442
BRIX1	4	0.000571	0.013264	0.000716	0.015062
ARPP19	7	0.000612	0.013849	0.000675	0.014483
CCND3	8	3.70E-07	0.000145	0.00198	0.028327
GSKIP	2	0.001136	0.020011	0.000283	0.00863
HNRNPR	8	0.000842	0.016758	0.000498	0.011952
SLC39A11	2	0.000671	0.014548		
SLCO5A1	2	0.000671	0.014548		
C5orf24	4	0.000905	0.017552	0.000473	0.011556
GTF2H2	2	0.000742	0.015486	0.000616	0.01363
RBM4	13	0.000869	0.017032	0.000521	0.012146
VIM	9	0.000294	0.008833	0.0012	0.020593
NFIC	5	0.001025	0.018888	0.000426	0.010785
AGFG1	4	0.001052	0.019145	0.000422	0.010725
PDCD10	4	0.000701	0.015018		
ATF7IP2	5	0.001052	0.019145	0.000444	0.011057
ARGLU1	4	0.001023	0.018888	0.000474	0.011556
CCDC82	5	0.000727	0.015366		
TSN	6	0.001013	0.018859	0.000493	0.011906
OSBPL8	3	0.00019	0.00694	0.001559	0.02388
PARL	6	0.000429	0.011305	0.001117	0.019741
TNRC6A	6	0.001062	0.01925	0.000504	0.011976
RAB2A	6	0.001071	0.019299	0.0005	0.011952
MEF2C	8	0.000755	0.015682		
RPL10	8	0.000839	0.016739	0.000695	0.0147
ZNF136	3	0.000403	0.011036	0.001232	0.020776
GLRX	6	0.000804	0.016199	0.000759	0.01571
DDX54	7	0.001111	0.019694	0.00053	0.012258
MTF2	6	0.001077	0.019365	0.000561	0.012805
DGKZ	13	0.001029	0.018888	0.000597	0.013328
IKZF5	3	0.001315	0.021979	0.0004	0.0103
RARA	6	0.001177	0.020337	0.000517	0.012082
LINS	3	0.001197	0.020495	0.00051	0.011976
C1D	4	0.0008	0.016164	0.000826	0.016619
HES6	2	0.000824	0.016528	0.00081	0.016377

Gene Name	Number of Isoforms	Type 1 Screening		Type 2 Screening	
		LRT p-value	FDR	LRT p-value	FDR
SSBP2	7	0.000899	0.017542	0.00074	0.015423
CEP63	6	0.00082	0.016483	0.000849	0.016904
RC3H1	3	0.001044	0.019082	0.000674	0.014483
BIRC5	4	0.000786	0.016009	0.000915	0.017622
PTPN12	5	0.000417	0.011123	0.001425	0.022577
RPL5	5	0.000741	0.015486	0.000988	0.018272
UHRF2	7	0.000846	0.01676	0.00086	0.017
TIPARP	5	0.000766	0.015728	0.000976	0.018105
DDX60L	7	0.000507	0.012395	0.001307	0.021545
PHF8	4	0.00086	0.016946	0.000869	0.01713
LEO1	2	0.001561	0.024766	0.000329	0.009434
RBM5	13	0.001168	0.020258	0.000656	0.014307
PEPD	2	0.000511	0.012416	0.001379	0.022263
SLC2A5	5	0.000264	0.008379	0.001809	0.026521
NCAPD3	2	0.001616	0.025377	0.00034	0.009542
FAM188A	3	0.000912	0.017552		
METTL23	4	0.000913	0.017552		
CUX1	8	0.000912	0.017552		
POGZ	14	0.001163	0.020248	0.000706	0.014893
ARID1B	5	0.000715	0.01519	0.001151	0.020017
SEL1L3	4	0.000923	0.017609		
SLC2A3	6	0.001345	0.022075	0.000585	0.013197
RNF6	5	0.001323	0.02199	0.000598	0.013328
CNOT2	10	0.001015	0.018864	0.000816	0.016466
MIS12	2	0.000912	0.017552	0.000935	0.017797
NOL8	6	0.000889	0.017376	0.000975	0.018105
KIDINS220	5	0.001354	0.022119	0.000606	0.013473
HECTD1	10	0.001113	0.019694	0.000789	0.016099
SPINK2	3	0.000101	0.004467	0.002431	0.0314
MAN1A1	2	0.000944	0.017974		
MGST1	6	0.000978	0.018344	0.000947	0.017928
CHD2	13	0.001023	0.018888	0.000891	0.017472
NFKBIA	9	0.001211	0.02063	0.000778	0.016033
MRI1	2	0.001765	0.026854	0.00037	0.00991
TAGLN	3	0.001165	0.020248	0.000845	0.016872
TRIM23	2	0.001234	0.020898	0.000803	0.016298
RNFT1	4	0.000585	0.013489	0.001554	0.023856
SENP6	9	0.000418	0.011123	0.001788	0.026299
SH3KBP1	4	0.001433	0.023107	0.000666	0.01442
VPS16	3	0.00079	0.016054	0.001315	0.021545
USP25	4	0.001007	0.018831		
SNX10	2	0.001009	0.018831		
INO80	3	0.00165	0.025632	0.000523	0.012146
MED15	14	0.001318	0.021979	0.00079	0.016099

Gene Name	Number of Isoforms	Type 1 Screening		Type 2 Screening	
		LRT p-value	FDR	LRT p-value	FDR
KLF6	6	0.001481	0.023682	0.000667	0.01442
SORBS3	7	0.000919	0.017609	0.001213	0.020602
NFAT5	4	0.000966	0.018258	0.001147	0.020017
TRIT1	2	0.001213	0.020631	0.000925	0.01768
IER3IP1	2	0.001919	0.02806	0.000408	0.010428
HSPH1	4	0.000323	0.00944	0.002142	0.029265
CUL5	6	0.000339	0.009729	0.002119	0.029123
ABHD3	4	0.000515	0.012468	0.001822	0.026656
CLN8	2	0.001092	0.019563		
RPUSD1	4	0.001652	0.025632	0.00061	0.013518
CEP85L	3	0.001776	0.026922	0.00054	0.012431
RPS6KA1	7	0.001108	0.019691		
NPTN	4	0.000907	0.017552	0.001347	0.021914
XPNPEP1	11	0.001309	0.021922	0.000912	0.017621
PTPMT1	4	0.001631	0.025468	0.000646	0.014158
FBXO9	7	0.001187	0.020399	0.00106	0.019243
POLG2	3	0.000978	0.018344	0.001285	0.021301
CNOT1	11	0.001178	0.020337	0.001085	0.019347
PLIN2	7	0.001133	0.020011		
REL	2	0.001982	0.028816	0.000468	0.011494
ATP5A1	12	0.001094	0.019563	0.00123	0.020776
PIGK	2	0.001163	0.020248		
PCBP2	18	0.001163	0.020248		
CRAMP1L	4	0.00116	0.020248		
ZEB1	5	0.001157	0.020248		
SLAIN2	5	0.001195	0.020495		
ZNF143	3	0.0012	0.020499		
WDR41	8	0.001326	0.02199	0.001067	0.019243
TRH	2	2.99E-05	0.002051	0.003518	0.039226
SMYD3	5	0.001183	0.020389	0.001301	0.021533
STX8	4	0.001853	0.027651	0.00068	0.014495
BMI1	3	0.00107	0.019299	0.001465	0.02298
GTF3C2	5	0.001774	0.026922	0.000741	0.015423
TFEC	3	0.001907	0.028033	0.000678	0.014493
GRIPAP1	6	0.001724	0.026547	0.000777	0.016033
HGS	10	0.001621	0.025416	0.000898	0.017526
WIPI2	11	0.001476	0.023661	0.001085	0.019347
SIK3	13	0.001628	0.025466	0.000926	0.01768
TNFAIP2	5	0.00129	0.021695		
LYRM5	4	0.001883	0.027901	0.000748	0.015521
SEC31A	17	0.001355	0.022119	0.001309	0.021545
VMA21	2	0.002189	0.030409	0.000593	0.013294
KLF7	5	0.001859	0.027651	0.000782	0.016065
UBA2	6	0.001309	0.021922		

Gene Name	Number of Isoforms	Type 1 Screening		Type 2 Screening	
		LRT p-value	FDR	LRT p-value	FDR
GGCT	2	0.001712	0.026416	0.000888	0.017459
HMGN5	2	0.001327	0.02199		
CAMSAP1	3	0.001345	0.022075		
SH3BP5	3	0.001346	0.022075		
SH3BP2	9	0.00143	0.023107	0.001319	0.021559
ARF4	6	0.001867	0.027708	0.00086	0.017
TMEM167A	5	0.001583	0.024967	0.00112	0.019747
BECN1	9	0.001063	0.01925	0.00174	0.026075
CEP44	2	0.002441	0.032804	0.000549	0.012572
NUP133	4	8.52E-05	0.004012	0.003889	0.041638
TMEM123	5	0.001423	0.023047		
CDK16	12	0.001823	0.027484	0.001016	0.018624
RPS13	9	0.000922	0.017609	0.002016	0.028531
TLK1	6	0.001897	0.027967	0.000993	0.018333
SYNJ1	2	0.001454	0.023353		
GFM2	3	0.001453	0.023353		
PLK4	3	0.001829	0.027484	0.001057	0.019241
RPL30	11	0.001825	0.027484	0.001065	0.019243
CYCS	4	0.002001	0.028873	0.000947	0.017928
HNRNPA3	4	0.002183	0.030409	0.000831	0.016637
CLDND1	8	0.001232	0.020898	0.00178	0.026267
SCOC	3	0.000327	0.009506	0.003298	0.037794
RPP30	6	0.001752	0.026774	0.001215	0.020602
METTL14	2	0.002475	0.032986	0.000688	0.014604
RPUSD4	6	0.002133	0.030118	0.000952	0.017955
MTRF1L	5	0.00222	0.030488	0.000916	0.017622
NPM1	10	0.002004	0.028873	0.001071	0.01927
SEPP1	2	0.001511	0.0241		
GNPTAB	5	0.002172	0.030376	0.000975	0.018105
SZRD1	6	0.001831	0.027484	0.001271	0.021113
PPIP5K2	5	0.00164	0.02555	0.001489	0.023213
CUL4B	3	0.001542	0.024514		
LTBP3	12	0.001942	0.028335	0.001256	0.021
SIKE1	2	0.002786	0.034862	0.000675	0.014483
MBP	4	0.002193	0.030409	0.001084	0.019347
TAGAP	3	0.001979	0.028816	0.001251	0.020971
WNK1	8	0.001382	0.02252	0.001888	0.02727
PPP1R3E	4	0.002382	0.032296	0.000901	0.017539
PAPD4	10	0.002118	0.030036	0.00114	0.019969
PNPLA8	6	0.002107	0.030018	0.001153	0.020017
CDKN1B	3	0.00159	0.025019		
CEP170	9	0.001762	0.026854	0.001492	0.023213
TGFBR1	4	0.002439	0.032804	0.000905	0.017539
FBXL3	3	0.001912	0.028044	0.001399	0.02235

Gene Name	Number of Isoforms	Type 1 Screening		Type 2 Screening	
		LRT p-value	FDR	LRT p-value	FDR
LUC7L3	11	0.000349	0.009918	0.003726	0.040593
C19orf53	5	0.002119	0.030036	0.001209	0.020602
MTMR6	2	0.002242	0.03071	0.001137	0.019945
PMPCB	7	0.002186	0.030409	0.001189	0.020448
TIMM10B	3	0.001095	0.019563	0.002421	0.031325
PTGER4	4	0.002167	0.030376	0.001237	0.020776
CSE1L	3	0.001288	0.021695	0.002174	0.029489
MTRR	4	0.001419	0.023021	0.001983	0.028327
RASA1	2	0.002003	0.028873	0.00142	0.022539
SRSF9	5	0.002123	0.030036	0.00131	0.021545
SVIL	2	0.00252	0.033326	0.001008	0.018524
SHOC2	4	0.002093	0.029879	0.001384	0.022296
LAMA5	3	0.001689	0.026154		
FAM117B	2	0.0027	0.034345	0.000958	0.017991
SLC12A6	7	0.001336	0.022075	0.002261	0.030303
GNA12	5	0.002222	0.030488	0.001345	0.021914
ZBTB44	5	0.002304	0.031401	0.00126	0.021027
ANXA7	6	0.002459	0.032874	0.001107	0.019598
GOLT1B	3	0.002734	0.03455	0.000948	0.017928
FAM160B1	3	0.002753	0.034578	0.000958	0.017991
DENND3	18	0.002214	0.030488	0.001377	0.022263
PREX1	4	0.002092	0.029879	0.001454	0.022944
KARS	4	0.00268	0.034345	0.001005	0.018501
BRWD3	2	0.002169	0.030376	0.001414	0.022533
BTBD3	3	0.002883	0.035565	0.000905	0.017539
ARID5A	5	0.002573	0.033763	0.001095	0.019446
MRPL13	3	0.002877	0.035549	0.000921	0.017677
M6PR	9	0.001838	0.027547	0.001706	0.025732
PPP1CB	6	0.001847	0.027624	0.001699	0.025666
PAQR3	2			0.001831	0.026687
ARHGAP21	2	0.001738	0.026709		
SERPING1	5	0.001741	0.026709		
FAM160A2	4	0.001579	0.024951	0.002028	0.028601
PHF11	9	0.001809	0.027371	0.001768	0.026211
ANP32A	7	0.001892	0.027967	0.001694	0.025646
GBP2	4	0.001042	0.019082	0.002895	0.034629
TTC14	4	0.000483	0.01199	0.003924	0.041784
RPL13	12	0.002269	0.030977	0.00146	0.022951
VMP1	11	0.002162	0.030376	0.001542	0.023752
KIAA1143	2			0.001881	0.027208
SEL1L	3	0.000757	0.015682	0.003452	0.038761
GORAB	2	0.000537	0.012812	0.003913	0.041723
DEF6	4	0.002595	0.033968	0.001204	0.020602
CREBZF	5	0.002621	0.034081	0.001216	0.020602

Gene Name	Number of Isoforms	Type 1 Screening		Type 2 Screening	
		LRT p-value	FDR	LRT p-value	FDR
KIF20B	4	0.002868	0.035507	0.001074	0.019277
NDUFA4	6	0.002693	0.034345	0.001213	0.020602
CRCP	3	0.002548	0.033552	0.001316	0.021545
SHPRH	3	0.002451	0.032822	0.001418	0.022539
WDR33	5	0.00134	0.022075	0.002703	0.033419
KLHL2	6	0.001281	0.021646	0.00283	0.034178
SMCHD1	7	0.002093	0.029879	0.001733	0.026075
PMS1	3			0.001955	0.028019
TCERG1	9	0.002449	0.032822	0.001496	0.023231
JAG1	2	0.00356	0.040023	0.000786	0.016099
UBE4B	5	0.002652	0.034293	0.001355	0.021964
ZFP36L1	4	0.002869	0.035507	0.001236	0.020776
ABCE1	3	0.00317	0.037347	0.001042	0.019019
ZC3H14	8	0.002547	0.033552	0.00145	0.022926
STARD7	2			0.001992	0.02835
FAM208A	6	0.001858	0.027651	0.002098	0.029065
HOPX	4	0.000607	0.013848	0.004098	0.043117
LYAR	2			0.002024	0.0286
ALG13	8	0.00269	0.034345	0.00146	0.022951
LRRC47	2			0.002039	0.02865
METTL17	3	0.000563	0.013218	0.004275	0.044142
ITM2A	4	0.003077	0.036961	0.001186	0.020442
BICD2	2			0.002061	0.028739
CAPN3	2			0.00206	0.028739
PPP1R12B	3			0.002076	0.028856
EMD	8	0.001996	0.028873		
RNF168	2	0.00374	0.041457	0.000828	0.016619
NUP54	7	0.002214	0.030488	0.00192	0.02762
SLC30A5	5	0.00031	0.009144	0.005163	0.048987
ZMAT2	2	0.001703	0.026321	0.002471	0.031864
TMEM64	2	0.003596	0.040179	0.000976	0.018105
RASA2	2	0.002936	0.035999	0.001393	0.022336
TXN	3	0.003366	0.038779	0.001124	0.01977
CLEC2B	4	0.002164	0.030376	0.001996	0.02835
FLI1	3			0.002167	0.029459
COX7A2	5	0.003102	0.036961	0.001354	0.021964
GOLPH3	2	0.003826	0.04209	0.000851	0.016904
PCGF1	5	0.002053	0.029514		
CANT1	2	0.002484	0.033024	0.001794	0.026349
CASK	2	0.002074	0.029765		
WRN	2	0.002214	0.030488	0.002105	0.029073
CCT2	12	0.002422	0.032715	0.001846	0.026853
L3MBTL3	2			0.002201	0.029805
GAPT	6	0.002597	0.033968	0.001752	0.026122

Gene Name	Number of Isoforms	Type 1 Screening		Type 2 Screening	
		LRT p-value	FDR	LRT p-value	FDR
SLC9A6	2	0.002623	0.034081	0.001746	0.026075
ARL6IP6	3			0.002237	0.030082
CAD	5			0.002241	0.030082
CD9	2	0.003105	0.036961	0.001491	0.023213
ATXN7	7	0.003074	0.036961	0.0015	0.023233
ELOVL5	4	0.002175	0.030376		
HNRNPC	21	0.002727	0.034547	0.001772	0.026221
ITSN2	5	0.001398	0.022728	0.003338	0.038122
CAPZA1	6	0.003096	0.036961	0.001563	0.023892
GMNN	2	0.000458	0.011658	0.005277	0.049288
ATAD2	5			0.002288	0.030543
ZNF333	3			0.002302	0.030543
BRIP1	3	0.003575	0.040023	0.001267	0.021095
LAS1L	5			0.002325	0.030591
AGO3	5			0.00232	0.030591
CDKAL1	2			0.00231	0.030591
ABCG1	4			0.002314	0.030591
RPS6KB1	6	0.002475	0.032986	0.002002	0.028385
CPSF3	3	0.003234	0.037846	0.001527	0.023575
IKBKAP	3	0.002251	0.030781		
PCF11	4	0.003307	0.038381	0.001502	0.023233
LANCL2	3			0.002352	0.030839
CSNK1G3	4			0.002372	0.030941
SREK1	11	0.002669	0.034345	0.001919	0.02762
LPP	3			0.002386	0.031075
ZNF800	4	0.000586	0.013489	0.005091	0.048773
OPTN	4	0.002932	0.035998	0.001782	0.026267
ZWINT	6	0.001915	0.028044	0.002909	0.034704
PPFIA1	5	0.002649	0.034293	0.002032	0.028608
C3orf17	6	0.002324	0.031598		
RAD51AP1	2	0.002327	0.031598		
NEK6	2	0.004164	0.044541	0.00104	0.019017
RHOH	4	0.002698	0.034345	0.002133	0.029214
BAZ2B	10	0.003199	0.037573	0.001745	0.026075
SAFB2	8	0.003301	0.038363	0.001674	0.025386
SEC14L1	9	0.002522	0.033326	0.0023	0.030543
USP47	7	0.002748	0.034578	0.002163	0.029459
NSMAF	5	0.002894	0.035655	0.002048	0.028722
MARK2	5			0.002528	0.032437
ACP1	6	0.00357	0.040023	0.00164	0.024919
TNFRSF25	2			0.002537	0.032496
TMEM138	8	0.002	0.028873	0.003098	0.036187
CCDC115	2			0.002548	0.032534
NAA30	3	0.003417	0.03908	0.001766	0.026211

Gene Name	Number of Isoforms	Type 1 Screening		Type 2 Screening	
		LRT p-value	FDR	LRT p-value	FDR
DEDD	4			0.002564	0.032646
LTBP4	3			0.00257	0.032659
GAS7	5	0.002418	0.032715		
TSPAN3	8			0.002582	0.032748
ARL6IP5	3	0.002435	0.032804		
TCF12	5	0.003722	0.041418	0.001589	0.024245
TIMP1	5	0.003743	0.041457	0.001592	0.024245
U2AF1	10	0.003372	0.038786	0.001873	0.027148
NHSL1	2	0.002487	0.033024		
PPP2R3C	10	0.002492	0.033038		
DDX20	3	0.003555	0.040023	0.001741	0.026075
MBIP	3	0.003067	0.036959	0.002135	0.029214
TMEM57	2			0.002635	0.0331
CPNE3	3	0.001099	0.019578	0.004688	0.046743
EFCAB14	2			0.002655	0.033199
MASTL	3	0.002919	0.035902	0.002298	0.030543
ARF1	11	0.00212	0.030036	0.003129	0.036449
USP9X	4			0.002671	0.033266
C12orf45	2	0.004174	0.044541	0.001388	0.022303
DCAF8	10	0.003278	0.038166	0.002064	0.028739
TMEM126A	2	0.004398	0.04649	0.001182	0.02042
PEX2	4			0.002716	0.033532
PAIP2	6	0.002976	0.036243	0.00237	0.030941
OXR1	3	0.004007	0.043544	0.001548	0.023805
SLU7	5			0.002744	0.033719
SACS	3			0.00275	0.033732
PHC3	8			0.002755	0.033747
ITPR1	4	0.00257	0.033763		
CCDC14	7	0.00175	0.026774	0.00378	0.040962
UBXN4	6			0.002773	0.033881
GPATCH2L	5			0.002779	0.033881
AKIRIN1	3	0.002602	0.033972		
MAP3K5	2	0.002612	0.034053		
OSBP	3			0.002804	0.034075
MEPCE	2			0.002808	0.034075
RASSF2	3	0.002627	0.034081		
PGD	6	0.003429	0.03916	0.002121	0.029123
SMC4	8	0.002714	0.034462	0.002775	0.033881
TRIM33	6	0.00285	0.035494	0.002608	0.032918
TOMM20	3	0.001028	0.018888	0.005388	0.049692
BRCA2	2	0.004831	0.049136	0.001096	0.019446
NSFL1C	7	0.003789	0.041798	0.001849	0.026853
PABPN1	6			0.002847	0.034335
IFT57	3	0.002674	0.034345		

Gene Name	Number of Isoforms	Type 1 Screening		Type 2 Screening	
		LRT p-value	FDR	LRT p-value	FDR
CEBPZ	2	0.002682	0.034345		
COMT	10	0.003392	0.038846	0.002215	0.029895
EIF3D	8			0.002858	0.034409
CNOT6L	6	0.002685	0.034345	0.002889	0.034629
PLSCR1	7	0.00273	0.034547		
AEBP2	4	0.002746	0.034578		
RARRES3	2	0.002758	0.034578		
FSCN1	4			0.00289	0.034629
MIB1	2			0.002898	0.034629
GIGYF2	7	0.003462	0.039481	0.002209	0.029864
PRKCI	2	0.003575	0.040023	0.002168	0.029459
GCC2	6			0.00292	0.034788
ENY2	12	0.003119	0.036961	0.002565	0.032646
IGLL1	3			0.002929	0.034839
RNF213	10	0.003666	0.040846	0.002106	0.029073
HSPBAP1	4	0.003035	0.036694	0.002664	0.033252
DAD1	3			0.002949	0.035026
MFF	11	0.003081	0.036961	0.002673	0.033266
RFX2	4			0.002964	0.035155
DDX27	4			0.002975	0.035172
FYN	6	0.003005	0.036437	0.002817	0.034135
SEPHS1	3	0.004171	0.044541	0.001758	0.026155
RBM6	9	0.002755	0.034578	0.003091	0.036187
ID2	3	0.003218	0.037736	0.002625	0.033031
YWHAZ	12	0.002983	0.036243	0.002885	0.034629
KLF13	3	0.001577	0.024951	0.004529	0.045921
TNFSF13	5	0.002859	0.035494		
C5orf22	2	0.00285	0.035494		
HEXB	5	0.002857	0.035494		
EOGT	4	0.001513	0.0241	0.00476	0.047034
MOAP1	2			0.003014	0.035582
CAPRIN1	8	0.003852	0.042245	0.002087	0.02896
RAB11A	6			0.00303	0.035713
CDC123	8	0.003747	0.041457	0.002324	0.030591
CLTC	6	0.003512	0.039757	0.002521	0.032406
TTLL5	4			0.003077	0.036163
CDK2	3			0.003092	0.036187
TLE4	9	0.003964	0.043309	0.002112	0.029108
CYB5R1	2	0.002984	0.036243		
TIAL1	11	0.00297	0.036243		
TAF4B	2	0.002974	0.036243		
RPS15	10	0.003819	0.042069	0.002282	0.03053
ARHGAP12	3	0.003009	0.036437		
USP8	6			0.003129	0.036449

Gene Name	Number of Isoforms	Type 1 Screening		Type 2 Screening	
		LRT p-value	FDR	LRT p-value	FDR
GRASP	7	0.003478	0.039543	0.002731	0.033612
ANKRD28	8	0.00313	0.037041	0.003093	0.036187
SAE1	3	0.004221	0.044982	0.001992	0.02835
S100PBP	4	0.003572	0.040023	0.002697	0.033406
RIOK1	3	0.003045	0.036762		
GOLGA7	6	0.002854	0.035494	0.00338	0.038392
CCSER2	3	0.003112	0.036961		
TNFSF13B	2	0.003114	0.036961		
TCF7L2	8			0.003193	0.037019
ORMDL1	7	0.003136	0.037041		
C15orf39	3	0.00314	0.037041		
CHRAC1	4	0.003112	0.036961	0.003227	0.037202
TOX4	5			0.003205	0.037107
PPP1R8	3			0.00322	0.037202
MAPK8IP3	8	0.004033	0.04369	0.002342	0.030752
CIR1	4	0.004029	0.04369	0.002368	0.030941
CMTM3	7	0.003342	0.038724	0.003052	0.035918
MIER3	4	0.00465	0.048035	0.00183	0.026687
ADAM15	14	0.004008	0.043544	0.002407	0.031196
PRKD3	3			0.003271	0.037553
C19orf24	2			0.003306	0.037829
C16orf80	5	0.001899	0.027967	0.004903	0.047839
YTHDC1	5			0.003341	0.038122
RFC1	5	0.00425	0.04511	0.002397	0.031163
BAZ1A	6	0.003268	0.03815		
C18orf25	4	0.003279	0.038166		
CNBP	6	0.003237	0.037846	0.003446	0.038761
DCTN4	7			0.003364	0.038327
COPS6	6	0.004447	0.046813	0.002222	0.02994
RMDN3	3			0.003378	0.038392
RIF1	5	0.004682	0.048065	0.002063	0.028739
AFTPH	6			0.003386	0.038405
PPIH	3			0.003391	0.03841
PTBP1	14	0.004059	0.043788	0.002652	0.033199
PSMD11	8	0.00441	0.046547	0.002296	0.030543
DDX24	8			0.003448	0.038761
HAUS3	3			0.003447	0.038761
XPC	2			0.00345	0.038761
TPD52	2	0.003356	0.038779		
CWC15	2	0.003365	0.038779		
TOB1	3	0.003378	0.038794		
UBA6	4	0.003384	0.03881		
PLXND1	12	0.004308	0.045597	0.002495	0.032119
MTSS1L	3			0.00347	0.038884

Gene Name	Number of Isoforms	Type 1 Screening		Type 2 Screening	
		LRT p-value	FDR	LRT p-value	FDR
PRRC1	4	0.004986	0.049877	0.001943	0.027897
CCNH	8			0.003494	0.039017
SH3BGRL	3			0.003492	0.039017
MOGS	5	0.002672	0.034345	0.00426	0.044056
TNPO1	6	0.003475	0.039543	0.003473	0.038884
JMJD1C	8	0.003738	0.041457	0.003227	0.037202
STX10	8	0.004631	0.047911	0.002401	0.031163
43895	3	0.003497	0.039695		
PRMT3	3	0.002201	0.030476	0.005144	0.048987
TNS3	3	0.00351	0.039757		
MKRN2	2			0.003591	0.039985
C1orf52	3			0.003605	0.039988
CTNNA1	9			0.003609	0.039988
MTDH	5			0.003617	0.039988
BTBD11	3			0.003606	0.039988
IL1RAP	9			0.003617	0.039988
PEX19	4	0.003547	0.040023		
IFIH1	2	0.003577	0.040023		
CEP152	4			0.003629	0.040051
CDCA4	2			0.003635	0.040051
SMARCD2	6			0.00364	0.040051
TP53INP1	3			0.003668	0.040224
TULP4	2			0.003669	0.040224
TROVE2	6	0.004669	0.048038	0.002544	0.032534
SEC63	8			0.003691	0.040348
MTMR1	2			0.00369	0.040348
MRPL9	6	0.004508	0.047094	0.00273	0.033612
SH3RF1	3	0.004674	0.048038	0.002586	0.032748
GEN1	2	0.004655	0.048035	0.002597	0.032843
SP1	3			0.003727	0.040593
SAP18	5			0.003729	0.040593
DOCK2	10	0.004548	0.047293	0.002827	0.034178
TACC1	12	0.004082	0.043954	0.003272	0.037553
MON2	6	0.004692	0.048105	0.00269	0.033406
IKZF4	2			0.003759	0.040804
EI24	4	0.003662	0.040846		
MORF4L2	7	0.004785	0.048857	0.002619	0.033012
BTBD7	6			0.003794	0.040962
ZDHHC13	2			0.003788	0.040962
IKBKE	3			0.003794	0.040962
ACSM3	5	0.002958	0.036208	0.004503	0.045792
FYB	5			0.00382	0.041183
SAR1A	8	0.004852	0.049287	0.002655	0.033199
IFITM1	3			0.003846	0.041345

Gene Name	Number of Isoforms	Type 1 Screening		Type 2 Screening	
		LRT p-value	FDR	LRT p-value	FDR
WSB1	6	0.004887	0.049454	0.002694	0.033406
DLC1	4			0.003873	0.041578
PSMA1	11			0.003895	0.041644
PLAU	3	0.004462	0.046813	0.003137	0.036488
RNF19A	6			0.003902	0.041663
EXOC5	3	0.003191	0.03754	0.004505	0.045792
RBM3	8	0.004896	0.049481	0.002801	0.034075
FRA10AC1	2			0.003929	0.041785
HIPK3	4			0.003947	0.041913
PBX2	11	0.004261	0.045165	0.003423	0.038722
PIK3R4	3	0.00409	0.043954	0.003643	0.040051
UBR3	3	0.004517	0.047107	0.003243	0.037331
SESN1	5	0.003853	0.042245		
BAX	9	0.003856	0.042245		
EIF3M	11	0.004102	0.043954	0.00374	0.040652
HMGXB4	4	0.004938	0.049522	0.002972	0.035172
SRPK2	4			0.004003	0.042453
DHX36	7	0.003768	0.041625	0.004149	0.043411
GSN	9			0.004026	0.042641
SURF1	2			0.004043	0.042663
SNX5	11			0.004044	0.042663
COA1	5			0.004038	0.042663
IL6ST	4	0.003102	0.036961	0.005183	0.049068
ITFG1	4	0.004913	0.049522	0.003156	0.036651
NFATC2IP	4			0.004099	0.043117
ABR	5	0.003944	0.04315		
POLR2J3	12			0.004108	0.043156
KIF5B	2			0.004122	0.043248
LY75	4			0.004135	0.043327
TRAPPC8	5	0.003359	0.038779	0.004958	0.047912
RNF166	7	0.004001	0.043544		
CCDC50	3			0.004188	0.043706
LTN1	3	0.004047	0.043735		
MRPL53	4			0.004198	0.043751
PXK	9			0.00421	0.043818
GABARAPL2	5			0.004221	0.043872
FAM107B	6			0.00423	0.043909
HCLS1	6	0.004099	0.043954		
SART3	5			0.004245	0.044011
TET2	7			0.004261	0.044056
STRAP	6	0.004479	0.046901	0.003829	0.041219
MAP3K7	3	0.00425	0.04511	0.004157	0.04344
TRIM8	3	0.003973	0.043348	0.004469	0.045661
ZDHHC18	2			0.004336	0.044716

Gene Name	Number of Isoforms	Type 1 Screening		Type 2 Screening	
		LRT p-value	FDR	LRT p-value	FDR
ZNF91	2	0.004662	0.048038	0.003882	0.041618
MAEA	9			0.00438	0.044986
ACTR3	6			0.004378	0.044986
RNF111	3			0.004375	0.044986
PTPN2	8	0.004248	0.04511		
SLC30A7	2			0.004422	0.045362
PSMD10	3			0.004432	0.045408
TUFM	4			0.004439	0.045413
NEK4	3			0.004482	0.045738
MKLN1	3			0.004497	0.045792
DNAJC4	6			0.004539	0.045962
SLC10A7	2			0.004582	0.045983
TRIAP1	2			0.004576	0.045983
AP1G1	10			0.004555	0.045983
BSCL2	4			0.004567	0.045983
DCP2	3			0.00458	0.045983
MYL6	15			0.004564	0.045983
FAM20B	2			0.004562	0.045983
DCUN1D5	5			0.004618	0.046221
ZNF460	2			0.004612	0.046221
WBP1	5	0.004094	0.043954	0.005203	0.049068
OGT	6			0.004659	0.046571
ZNF131	9			0.00467	0.046624
LCP2	7	0.004048	0.043735	0.005396	0.049692
GTF3C3	5			0.004702	0.046753
PFDN1	5			0.004713	0.046753
SEC22C	8			0.004706	0.046753
TIA1	7			0.004712	0.046753
MAP3K2	3			0.004729	0.046798
NCOA1	7			0.004724	0.046798
BCL11A	3	0.004456	0.046813		
DCTN3	4	0.004461	0.046813		
ATF1	2	0.004465	0.046813		
CHKB-CPT1B	2			0.004765	0.047034
LILRB2	4	0.00451	0.047094		
TMEM176A	3	0.004524	0.047118		
WDR43	5			0.004781	0.047125
UBAP1	7	0.004553	0.047293		
HOXA3	3	0.00457	0.047404		
WASF2	2			0.004825	0.047507
INPP4A	4			0.004842	0.047613
ZFAND2A	5	0.00487	0.049404	0.004516	0.045845
SUPT4H1	6			0.004853	0.047659
FAM120A	4			0.004869	0.04776

Gene Name	Number of Isoforms	Type 1 Screening		Type 2 Screening	
		LRT p-value	FDR	LRT p-value	FDR
FAM134C	2			0.00488	0.047804
DENND1A	6			0.004914	0.047839
ZBTB26	2			0.004904	0.047839
ZC3H11A	7			0.00491	0.047839
GTPBP1	8			0.004907	0.047839
VPS28	10			0.004928	0.047877
EXOSC10	7			0.00494	0.047877
CNOT6	2			0.004942	0.047877
ESYT1	8			0.00494	0.047877
YWHAB	5	0.004621	0.047877		
SGK3	4			0.004953	0.047912
SPATA13	4			0.005034	0.048579
NUDT5	5			0.005058	0.04863
PRPS1	3			0.005045	0.04863
C2orf47	3			0.005071	0.048705
HPS3	4			0.00508	0.048725
GRIK5	2	0.004773	0.048809		
FAM98B	2	0.004774	0.048809		
FAM49B	10			0.005101	0.048809
ATXN3	4			0.005111	0.048846
SHISA5	4			0.005149	0.048987
DESI2	2			0.005153	0.048987
SOCS5	2			0.005161	0.048987
ZNF197	2			0.005189	0.049068
MFN1	2			0.005198	0.049068
STXBP3	3			0.005195	0.049068
SPN	4	0.004933	0.049522	0.005054	0.04863
CENPU	4	0.004821	0.049136		
SSSCA1	4	0.004829	0.049136		
RNGTT	3			0.005261	0.049193
WDR52	2			0.005259	0.049193
SUMO3	5			0.005249	0.049193
ZCCHC17	2			0.005231	0.049193
SRSF6	2			0.005257	0.049193
KIAA1468	4			0.005257	0.049193
CSPP1	2			0.005247	0.049193
EIF5A	8	0.004945	0.049527	0.005132	0.048982
R3HDM2	8	0.004886	0.049454	0.005322	0.049532
CYB5R3	6	0.004935	0.049522		
MICB	2	0.004929	0.049522		
GPR124	2	0.00492	0.049522		
RELB	7			0.005318	0.049532
AMD1	6			0.005311	0.049532
USP7	11			0.005336	0.049606

Gene Name	Number of Isoforms	Type 1 Screening		Type 2 Screening	
		LRT p-value	FDR	LRT p-value	FDR
ZNF330	5			0.005345	0.049613
AASDHPPT	3			0.00535	0.049613
CD97	11			0.005359	0.049639
PMAIP1	3			0.005367	0.049655
ARF3	3			0.005386	0.049692
ELF4	2			0.005393	0.049692
SMC2	4			0.005426	0.049903
ISCU	7			0.005443	0.049909
TCEAL1	3			0.005443	0.049909
DDX11	6			0.005446	0.049909
SRPK1	6			0.005468	0.049937
NUMB	9			0.005455	0.049937
RABEP1	4			0.005467	0.049937
