# Supplementary material for: Two-step mixed model approach to analyzing differential alternative RNA splicing
Source: PLoS One. 2020 Oct 9;15(10):e0232646. doi: 10.1371/journal.pone.0232646 (PMC7546511; doi:10.1371/journal.pone.0232646)
Supplement: S4 Table — (PDF) [file pone.0232646.s013.pdf]

Supplementary Table 4. List of differentially expressed isoforms based on Type 1 screening test for AML Study.

Isoform ID	Gene Name	Fold Change	p-value (t test)	Two step significance threshold
ENST00000589978	UBE2S	-2.427633877	2.71E-05	0.002499361
ENST00000505804	TOPBP1	1.535638457	0.000103065	0.001666241
ENST00000492396	PDCD10	1.444896662	0.000752796	0.00124968
ENST00000558357	INO80	-1.532672827	0.000403874	0.001666241
ENST00000409386	CAMSAP1	-1.350314741	0.001149588	0.001666241
ENST00000338946	PHF8	1.412893887	9.48E-06	0.00124968
ENST00000438952	SYNJ1	1.509495198	0.00057238	0.002499361
ENST00000534766	PHF21A	1.404135988	0.00065312	0.000714103
ENST00000367505	SHPRH	1.234299914	0.000379352	0.001666241
ENST00000476559	RPGR	1.429731563	0.000408241	0.001666241
ENST00000473132	TOMM20	-1.775066087	0.001376212	0.001666241
ENST00000479630	MAP3K7	1.318308819	0.000357664	0.001666241
ENST00000585202	SRSF2	-2.297258612	0.000589028	0.00062484
ENST00000343505	NHSL1	1.160579649	0.000925395	0.002499361
ENST00000414822	CDKN1C	-1.565790215	5.41E-05	0.00124968
ENST00000263642	IFIH1	1.431828723	0.000939784	0.002499361
ENST00000462391	NPM3	-1.595652285	0.002002409	0.002499361
ENST00000468544	NPM3	-2.020812403	0.000645614	0.001666241
ENST00000400422	EIF4G3	1.475107133	0.000263469	0.000555414
ENST00000393063	DICER1	1.400462387	0.000183226	0.000714103
ENST00000493432	PLSCR1	1.455006852	0.000304003	0.000714103
ENST00000448205	PLSCR1	1.453313951	0.000731191	0.00083312
ENST00000441669	TRIT1	1.374795333	0.000533295	0.002499361
ENST00000566887	TMX3	1.298018111	7.55E-05	0.002499361
ENST00000299608	TMX3	1.270793049	0.000153875	0.004998722
ENST00000508610	MEF2C	1.684835914	0.000157714	0.00062484
ENST00000526109	FNBP4	2.065559696	6.93E-05	0.000499872
ENST00000379400	RASSF2	1.418507907	0.000558869	0.001666241
ENST00000368466	MAN1A1	-1.446332587	0.00024007	0.002499361
ENST00000325795	ORMDL1	1.451790507	0.000482707	0.000714103
ENST00000496647	EOGT	1.302686943	0.000516287	0.00124968
ENST00000476912	FAM188A	1.460726148	0.00124074	0.001666241
ENST00000448428	PRPF40A	-2.215019965	3.69E-05	0.000555414
ENST00000416400	VPS54	1.394934802	0.000547833	0.001666241
ENST00000409558	VPS54	1.323596909	6.28E-05	0.00124968
ENST00000382753	TMEM128	1.566303795	0.000218449	0.001666241
ENST00000503252	LPCAT1	1.641507219	6.30E-06	0.002499361
ENST00000383791	SH3BP5	1.485738242	0.000955094	0.002499361
ENST00000412806	SH3BP5	1.456406087	0.000466311	0.001666241
ENST00000305232	SIRT6	1.386699074	0.000156299	0.001666241
ENST00000493952	SATB1	1.959551927	5.74E-05	0.000714103

Isoform ID	Gene Name	Fold Change	p-value (t test)	Two step significance threshold
ENST00000372991	CCND3	1.761361992	0.001054096	0.00124968
ENST00000372987	CCND3	1.338443163	0.000997658	0.00118277
ENST00000414200	CCND3	1.625174381	8.93E-06	0.00062484
ENST00000510503	CCND3	1.536287891	0.000365888	0.00083312
ENST00000512426	CCND3	1.611346066	2.91E-05	0.000714103
ENST00000505064	CCND3	1.562073181	0.001510854	0.001666241
ENST00000476174	AZI2	1.418828491	0.000291146	0.00124968
ENST00000295748	AZI2	1.302516562	2.41E-05	0.000999744
ENST00000355845	MAP3K5	1.350572721	0.000963735	0.002499361
ENST00000497655	CYB5R1	1.160901186	0.001699259	0.002499361
ENST00000568067	TMEM87A	1.582607886	8.74E-05	0.000999744
ENST00000262895	GRIK5	-1.554824156	0.003636991	0.004998722
ENST00000301218	GRIK5	-1.403920322	0.002619174	0.003599822
ENST00000491510	TOP2B	1.491921058	0.000890808	0.000999744
ENST00000542520	TOP2B	1.520731651	0.000115469	0.00083312
ENST00000515770	DEK	1.707173281	8.00E-05	0.00083312
ENST00000244776	DEK	1.943880222	0.00015911	0.000999744
ENST00000358252	TNKS1BP1	-1.397650135	0.000260521	0.001666241
ENST00000511224	SEPP1	1.741552791	0.000945039	0.002499361
ENST00000507943	BOD1L1	1.441527384	0.000291133	0.000999744
ENST00000399878	SEL1L3	1.567981629	0.001243093	0.00124968
ENST00000497054	MOB1A	-1.646096151	0.001339208	0.001666241
ENST00000369751	IBTK	-1.223733255	0.000361183	0.00083312
ENST00000515125	GFM2	1.41799553	0.000480299	0.001666241
ENST00000559767	AQR	1.473404276	0.000235467	0.00124968
ENST00000523319	TPD52	1.409697077	0.001390257	0.002499361
ENST00000546904	PPP1CC	1.469007271	0.000483988	0.000555414
ENST00000261396	NUP133	1.294851672	0.000435528	0.001666241
ENST00000490352	NUP133	1.701919655	0.000339895	0.001300367
ENST00000521026	NDRG1	1.629292102	0.00035628	0.000555414
ENST00000436370	PEPD	1.572085079	0.000293154	0.002499361
ENST00000555705	EAPP	-1.245643831	0.001879508	0.002499361
ENST00000554361	PPP2R3C	1.806606937	0.00016857	0.000499872
ENST00000447319	HMGN5	1.467534439	0.004412726	0.004998722
ENST00000358130	HMGN5	1.278200259	0.002952481	0.003344561
ENST00000397901	CHMP1A	1.314708545	0.000661283	0.000964674
ENST00000253475	CHMP1A	-1.62666739	0.000685324	0.000999744
ENST00000478129	FNBP1	1.578507344	0.000157641	0.00083312
ENST00000379979	GLRX	1.457431781	0.000454568	0.00083312
ENST00000582501	POLG2	1.380398996	0.001086814	0.001666241
ENST00000335272	PITPNB	-1.650453519	0.000898587	0.001666241
ENST00000455418	PITPNB	1.460616754	0.00190538	0.002499361
ENST00000580104	TRAPPC8	1.309306841	0.000102409	0.000999744
ENST00000255559	SLC39A11	1.343196488	0.00224873	0.004998722

Isoform ID	Gene Name	Fold Change	p-value (t test)	Two step significance threshold
ENST00000542342	SLC39A11	1.355916366	0.000454271	0.002499361
ENST00000421484	PARL	1.482962815	0.000750917	0.00083312
ENST00000522635	TCEA1	1.79352929	0.001159015	0.001666241
ENST00000521086	TCEA1	-1.298581341	0.002090054	0.002499361
ENST00000373740	DSN1	1.487507867	4.39E-05	0.001666241
ENST00000413447	CYCS	-2.523672623	0.000213274	0.00124968
ENST00000475890	CSAD	1.436643031	7.45E-06	0.00124968
ENST00000565809	RPUSD1	1.356830538	0.001151238	0.00124968
ENST00000588705	IER3IP1	-1.676093611	0.002369722	0.002499361
ENST00000397137	BLCAP	1.798734544	1.81E-05	0.002499361
ENST00000418123	TRIP12	1.494645947	0.000377547	0.00041656
ENST00000536350	ACRBP	1.473899459	0.000313539	0.001666241
ENST00000558934	SPPL2A	1.617315826	3.38E-05	0.001666241
ENST00000559769	DMXL2	1.61374344	0.000586171	0.000714103
ENST00000323929	MRE11A	1.308617657	0.000202707	0.002499361
ENST00000564663	CCPG1	1.414348085	0.000559624	0.000714103
ENST00000392865	RGS10	-1.47476596	0.002216469	0.002499361
ENST00000311380	ARHGAP12	1.214002826	0.001130531	0.001666241
ENST00000278520	CCDC82	1.340344145	0.000226796	0.000999744
ENST00000439052	BNIP2	2.381825028	1.50E-05	0.000555414
ENST00000538155	ASUN	1.536805924	0.000479419	0.001666241
ENST00000482517	MYCBP2	1.580854101	3.42E-05	0.000714103
ENST00000482410	ZDHHC6	1.585282562	2.51E-05	0.001666241
ENST00000552606	CCDC59	1.441694299	0.000724018	0.00124968
ENST00000361236	TMEM123	1.252196909	0.000828925	0.001015805
ENST00000398136	TMEM123	1.547097666	0.001019773	0.00124968
ENST00000481438	DCTN3	1.303537236	0.001169515	0.00124968
ENST00000267229	RBM26	1.595546763	0.000435342	0.000999744
ENST00000579232	THOC1	1.464704055	0.000137296	0.000499872
ENST00000359785	PTPN22	1.604283874	1.13E-05	0.001334188
ENST00000528414	PTPN22	1.511297707	1.41E-05	0.001666241
ENST00000525799	PTPN22	1.39452347	0.000206932	0.002499361
ENST00000487510	ZRANB2	1.622594445	0.000491679	0.001666241
ENST00000477096	ZRANB2	1.505317157	0.000182363	0.00124968
ENST00000486374	ARHGAP21	1.370648735	0.000465782	0.002499361
ENST00000248701	SPINK2	2.366868487	0.001125108	0.001666241
ENST00000506738	SPINK2	1.988544065	0.003961034	0.004998722
ENST00000504762	SPINK2	2.336565699	0.002275416	0.002871515
ENST00000319410	KARS	-1.699910155	0.000178051	0.00124968
ENST00000460827	PCYT1A	1.42056201	0.000719273	0.001666241
ENST00000294119	UBXN1	-1.677830279	0.000248292	0.000418264
ENST00000532904	UBXN1	1.814664952	0.000257291	0.000433425
ENST00000529640	UBXN1	-1.940248878	0.00026976	0.000454429
ENST00000468222	MICU2	-1.625276475	0.000960484	0.000999744

Isoform ID	Gene Name	Fold Change	p-value (t test)	Two step significance threshold
ENST00000513216	CENPC	1.799320006	9.69E-06	0.000999744
ENST00000314062	DLG1	1.281888492	0.00040123	0.000714103
ENST00000489306	CEBPZ	1.495042698	0.00146919	0.002499361
ENST00000234170	CEBPZ	1.277301707	0.003696515	0.004998722
ENST00000370813	PIGK	1.528760815	0.000668019	0.002499361
ENST00000425080	LBR	-1.879429932	0.000800156	0.00083312
ENST00000524945	SLCO5A1	-1.343168394	0.00297348	0.004998722
ENST00000528658	SLCO5A1	-1.661323881	0.002125349	0.003572927
ENST00000439962	EDEM3	1.370608416	0.000339474	0.002499361
ENST00000318130	EDEM3	1.285042254	0.000821089	0.004998722
ENST00000521942	GTF2H2	-1.206212709	0.000634317	0.002499361
ENST00000494752	DPM1	1.643198668	0.000746956	0.00124968
ENST00000540661	MTMR6	-1.533148791	0.000871525	0.002499361
ENST00000329305	TPM2	1.534577874	0.00062387	0.002499361
ENST00000310864	IQCB1	1.314851193	0.000594856	0.001666241
ENST00000488117	C9orf72	1.375074318	0.000719104	0.00124968
ENST00000368346	ASH1L	1.283545809	0.001086908	0.00124968
ENST00000536305	SLC2A5	1.902780342	9.44E-05	0.000999744
ENST00000463593	NOL8	1.370057261	0.000498176	0.00083312
ENST00000370691	LAMA5	-1.298713245	0.000946697	0.002499361
ENST00000495695	LAMA5	-1.578561758	0.003066343	0.004998722
ENST00000252999	LAMA5	-1.349384049	0.000782077	0.002064748
ENST00000584687	ROCK1	1.455573179	0.000688932	0.000999744
ENST00000505301	ANKRA2	1.56620665	0.000778165	0.001666241
ENST00000506578	LEMD2	1.494037181	0.000325987	0.000555414
ENST00000428135	SNX13	1.271068424	0.000802922	0.00124968
ENST00000483056	FAM120AOS	1.451496066	0.000401144	0.00062484
ENST00000555932	MAX	1.884790356	0.000172238	0.00062484
ENST00000591000	POLR2J2	-2.085772325	2.40E-05	0.001666241
ENST00000479918	TMED5	-1.788339284	0.000762756	0.000999744
ENST00000449779	GSAP	1.241331257	0.000611143	0.002499361
ENST00000257626	GSAP	1.359024633	0.00034264	0.001666241
ENST00000376444	GRIPAP1	1.591658459	0.00060283	0.00083312
ENST00000540722	CWC15	1.385047174	0.000860359	0.002499361
ENST00000496489	NCK1	-1.796318653	5.59E-05	0.000714103
ENST00000467911	NCK1	-1.711900699	0.000192635	0.00083312
ENST00000573936	CHD3	1.49330774	0.000450099	0.000714103
ENST00000398243	S100PBP	-1.403211992	0.000246575	0.00124968
ENST00000356509	GMNN	1.386796152	0.000167501	0.002499361
ENST00000573759	MIS12	1.350356013	0.000385833	0.002499361
ENST00000380443	VPS16	1.283882121	0.000145116	0.001666241
ENST00000286824	TSPAN7	-1.489719799	0.000689708	0.001666241
ENST00000524711	EIF3M	1.597945336	0.000160135	0.000454429
ENST00000456692	RASA1	1.339516451	0.000813886	0.002499361

Isoform ID	Gene Name	Fold Change	p-value (t test)	Two step significance threshold
ENST00000591571	METTL23	1.361095366	0.00054695	0.00124968
ENST00000338517	SCOC	1.378904282	0.000595017	0.001666241
ENST00000487445	GCA	1.54583581	0.000125401	0.001666241
ENST00000460563	HMGN2	1.76407609	3.97E-05	0.00062484
ENST00000374166	RPS6KA1	1.228240264	0.000179095	0.000714103
ENST00000466153	TPP2	1.597084643	0.000392882	0.000714103
ENST00000354926	C7orf55-LUC7L2	-1.510336923	0.000270804	0.000714103
ENST00000263545	C7orf55-LUC7L2	1.748614515	0.000234239	0.00062484
ENST00000493765	TNFSF13B	1.504642311	0.000905407	0.002499361
ENST00000438229	OXR1	-1.372130812	0.0009542	0.001666241
ENST00000373247	FPGS	-1.574641525	0.000183651	0.000499872
ENST00000505618	RHOH	-1.651953636	0.000478183	0.00124968
ENST00000302649	TRH	-1.963021548	0.000481175	0.003448173
ENST00000507066	TRH	-2.838269026	0.000697547	0.004998722
ENST00000436959	KNTC1	1.306727541	0.000110501	0.00083312
ENST00000537348	KNTC1	1.311960449	9.17E-05	0.000714103
ENST00000361544	CDC14A	-1.364530614	0.000525577	0.000999744
ENST00000547146	DMTF1	1.226715042	0.000296677	0.000454429
ENST00000465148	CCNJ	-1.293933455	0.000186824	0.002499361
ENST00000435123	EEF1B2	-3.253894031	1.03E-05	0.00062484
ENST00000455150	EEF1B2	2.471995061	0.000626027	0.000714103
ENST00000420397	43897	1.505008024	0.000363509	0.00124968
ENST00000485687	TBC1D23	-1.623437742	3.37E-05	0.000714103
ENST00000440940	MTRR	1.214725459	0.000801074	0.00124968
ENST00000436146	RPL15	4.052658639	0.000160515	0.000714103
ENST00000477649	VMA21	-2.051292792	0.000541108	0.002499361
ENST00000514343	PTGER4	-2.09296527	0.001246286	0.00124968
ENST00000510872	43896	-1.805485412	0.000550288	0.000555414
ENST00000415167	NPR3	-1.722913873	0.000658054	0.00124968
ENST00000457869	SERPING1	1.522259183	0.002141356	0.002686626
ENST00000340687	SERPING1	1.459296235	0.003984195	0.004998722
ENST00000378323	SERPING1	1.977602121	0.000127232	0.000999744
ENST00000378324	SERPING1	1.708346674	0.001655924	0.002077585
ENST00000531133	SERPING1	1.594808974	0.001058382	0.001327887
ENST00000515798	BRIX1	1.2065408	0.000740506	0.00124968
ENST00000371436	RUNX2	-1.630822141	0.000190921	0.00083312
ENST00000456351	MICB	1.300059165	0.001519118	0.002499361
ENST00000521271	CPNE3	1.506374824	0.000194898	0.001666241
ENST00000553331	PNN	-1.633152455	0.000269256	0.00124968
ENST00000349457	GPR114	1.543254804	0.001210477	0.00124968
ENST00000580465	BPTF	1.306974168	0.000448027	0.00062484
ENST00000578121	TAF4B	-1.224883721	0.001714156	0.002499361
ENST00000580499	RNF138	-2.075589391	0.000315097	0.000999744
ENST00000412756	TTC14	1.407234777	0.000180718	0.00124968

Isoform ID	Gene Name	Fold Change	p-value (t test)	Two step significance threshold
ENST00000527628	EI24	1.469853332	0.000807294	0.00124968
ENST00000352618	RAD51AP1	1.334790353	0.000723107	0.002499361
ENST00000427956	TXNRD1	-1.697822233	9.09E-07	0.000555414
ENST00000332180	KIAA1033	1.345100444	0.000267406	0.001666241
ENST00000338523	SNX10	1.544068082	0.001520421	0.004998722
ENST00000396376	SNX10	1.376349782	0.001108454	0.003644291
ENST00000374990	TGFBR1	1.296727007	0.000238078	0.00124968
ENST00000261308	PPWD1	1.270186515	2.35E-05	0.000714103
ENST00000520991	CLN8	-1.527540534	0.000243447	0.002499361
ENST00000533359	MED17	-1.601746792	0.000297561	0.000499872
ENST00000409824	SP100	1.567897235	0.000448463	0.000454429
ENST00000409112	SP100	1.429797492	7.94E-05	0.000384517
ENST00000452345	SP100	1.671080956	1.35E-05	0.000357052
ENST00000431952	SP100	1.487693166	0.000228878	0.00041656
ENST00000398864	AEBP2	-1.549761754	0.000180004	0.00124968
ENST00000498600	GORAB	1.258018622	0.000671315	0.002499361
ENST00000397412	CRAMP1L	-1.307914305	0.000673341	0.00124968
ENST00000555443	KLHDC2	1.2801052	0.00057491	0.000714103
ENST00000557063	KLHDC2	1.276176153	0.00073476	0.00083312
ENST00000463551	SLC25A5	1.721682872	0.000236798	0.00124968
ENST00000464658	RPS8	1.795078714	0.000530023	0.00062484
ENST00000297661	UBAP1	-1.497647043	0.000284278	0.000714103
ENST00000471350	COMMD3	1.585985979	0.00028173	0.000499872
ENST00000463688	COMMD3	1.55159866	0.000366332	0.000555414
ENST00000376552	TLE4	-1.414465902	0.000214432	0.000555414
ENST00000570163	ATF7IP2	1.893709284	1.70E-05	0.000999744
ENST00000511158	SLC30A5	1.389642715	6.35E-05	0.000999744
ENST00000338592	CRCP	-1.491642909	0.001483404	0.001666241
ENST00000490422	ZMYM2	1.786494259	0.000225267	0.000714103
ENST00000549546	TGIF1	-1.812195068	0.0003403	0.000555414
ENST00000253925	PPFIA1	1.265486768	0.000484428	0.000999744
ENST00000377967	KDM6A	1.34398397	0.00051542	0.000714103
ENST00000480388	AKAP2	-1.429503646	3.51E-05	0.002499361
ENST00000374525	AKAP2	-1.546854267	0.001348236	0.004998722
ENST00000549206	SOCS2	2.01290949	0.000160238	0.00083312
ENST00000549122	SOCS2	1.626052089	0.000540803	0.00124968
ENST00000551883	SOCS2	1.977143135	0.000402064	0.000999744
ENST00000262126	ANKRD12	1.562350689	0.000101524	0.000999744
ENST00000463980	ITPR1	1.366116785	0.000147486	0.00124968
ENST00000506824	KLHL2	1.26351022	0.00027879	0.00083312
ENST00000537871	RARRES3	-1.363019569	0.001083307	0.002499361
ENST00000483137	HNRNPA3	2.232194213	0.000230904	0.00124968
ENST00000380771	WRNIP1	1.381280639	0.001375939	0.001666241
ENST00000262053	ATF1	1.306481532	0.001317064	0.002499361

Isoform ID	Gene Name	Fold Change	p-value (t test)	Two step significance threshold
ENST00000432286	TNRC6A	1.482031809	0.000529831	0.00083312
ENST00000438834	BZW2	-1.547720368	0.000259645	0.000454429
ENST00000452975	BZW2	-1.757608122	0.000438213	0.000499872
ENST00000394335	MST4	1.520856681	0.000133117	0.000999744
ENST00000554703	NAA30	-1.374659497	0.001348623	0.001666241
ENST00000553389	METTL17	1.479945078	0.000213358	0.001666241
ENST00000248594	PTPN12	1.375449707	0.00069072	0.000999744
ENST00000307305	PHTF2	1.261118183	0.000146491	0.00124968
