# Supplementary material for: Two-step mixed model approach to analyzing differential alternative RNA splicing
Source: PLoS One. 2020 Oct 9;15(10):e0232646. doi: 10.1371/journal.pone.0232646 (PMC7546511; doi:10.1371/journal.pone.0232646)
Supplement: S5 Table — (PDF) [file pone.0232646.s014.pdf]

Supplementary Table 5. List of differentially expressed isoforms based on Type 2 screening test for AML Study.

Isoform ID	Gene Name	Fold Change	p-value (t test)	Two step significance threshold
ENST00000589978	UBE2S	-2.427633877	2.71E-05	0.002499361
ENST00000505804	TOPBP1	1.535638457	0.000103065	0.001666241
ENST00000466253	DCAF8	-1.027623821	0.819595137	0.004998722
ENST00000490546	PUM1	-1.100253648	0.297598798	0.002704675
ENST00000353088	NSFL1C	1.044918026	0.615811714	0.004663019
ENST00000302345	CANT1	-1.035813151	0.699581722	0.004998722
ENST00000533397	RPL8	-1.064600846	0.687516225	0.00367696
ENST00000418304	ARID4B	-1.275573963	0.063152115	0.00083312
ENST00000531008	RPS13	1.318320272	0.014799376	0.000555414
ENST00000394094	SENP7	1.244368346	0.007130122	0.00124968
ENST00000370094	MGEA5	-1.0374243	0.759170792	0.004998722
ENST00000526601	ZNF195	-1.12917352	0.201615397	0.001858206
ENST00000264538	IFT57	1.239057817	0.003849236	0.001666241
ENST00000343455	DICER1	1.068763734	0.289038672	0.002499361
ENST00000532120	EIF4G2	1.269846611	0.335909324	0.002769731
ENST00000519583	CLK4	1.201224318	0.044759588	0.000999744
ENST00000530207	FNBP4	1.140768377	0.247352742	0.001237614
ENST00000504813	CNBP	1.095340792	0.412817572	0.002071455
ENST00000425534	EIF4E3	1.229898234	0.075539402	0.004998722
ENST00000283415	LPCAT1	1.146445424	0.120416594	0.004998722
ENST00000483316	BAZ2B	-1.058717652	0.195002515	0.001051354
ENST00000343439	BAZ2B	-1.022536131	0.809782621	0.004365934
ENST00000409784	RAB1A	-1.047774757	0.573127301	0.004998722
ENST00000452260	SATB1	1.216464077	0.021522717	0.001073818
ENST00000409351	BCL11A	1.417807438	0.013310228	0.002499361
ENST00000473731	KIDINS220	1.296684153	0.002685595	0.000999744
ENST00000319688	KIDINS220	-1.18666031	0.005912766	0.00124968
ENST00000418530	KIDINS220	1.024885005	0.715635039	0.004998722
ENST00000427836	PLEKHM3	1.25145566	0.008989259	0.00124968
ENST00000447645	PLEKHM3	1.089932194	0.150912159	0.002499361
ENST00000457206	PLEKHM3	-1.045822499	0.351123452	0.004998722
ENST00000536863	DCAF16	-1.514620205	0.004613058	0.001666241
ENST00000510783	RFC1	1.270703898	0.021488183	0.000999744
ENST00000381897	RFC1	-1.085218426	0.093490942	0.00124968
ENST00000509084	RFC1	1.012596331	0.916996488	0.004998722
ENST00000409969	MOB1A	1.211728937	0.138704488	0.002499361
ENST00000505927	RICTOR	1.353657712	0.008843889	0.000999744
ENST00000503400	IBTK	-1.018734822	0.768088554	0.004998722
ENST00000485752	RNF149	1.311421467	0.017418201	0.000927647
ENST00000354184	MTSS1	-1.245197588	0.105895692	0.00124968
ENST00000557369	HECTD1	-1.015310493	0.911108908	0.004998722

Isoform ID	Gene Name	Fold Change	p-value (t test)	Two step significance threshold
ENST00000300619	ZNF91	-1.059115207	0.370209253	0.004998722
ENST00000549271	DDX54	1.13673814	0.177616969	0.001279039
ENST00000314045	DDX54	-1.062659167	0.391696249	0.002820647
ENST00000573895	ABR	1.148781176	0.115405619	0.004998722
ENST00000555629	NFKBIA	1.134286313	0.382722235	0.001941983
ENST00000507605	GLRX	1.00588162	0.933150448	0.004998722
ENST00000320996	PITPNB	1.123918498	0.219277875	0.004998722
ENST00000545952	SS18	-1.228712504	0.023371459	0.001666241
ENST00000422087	PNPLA8	-1.114318175	0.217546425	0.00168069
ENST00000391836	SMYD3	1.09141424	0.631562186	0.004998722
ENST00000489372	JMJD1C	-1.105910656	0.442590564	0.002263025
ENST00000427635	HERC4	-1.084968433	0.118414742	0.001666241
ENST00000518211	RB1CC1	-1.196704731	0.097168604	0.002063093
ENST00000317096	PARL	-1.016347517	0.883437995	0.004960548
ENST00000522635	TCEA1	1.79352929	0.001159015	0.001666241
ENST00000565809	RPUSD1	1.356830538	0.001151238	0.00124968
ENST00000530769	TAF1D	1.040123679	0.628310768	0.003197547
ENST00000540232	TAF1D	1.036624036	0.769077033	0.003913922
ENST00000528734	TAF1D	1.170841563	0.250363801	0.00127413
ENST00000536350	ACRBP	1.473899459	0.000313539	0.001666241
ENST00000315141	LEO1	-1.315974908	0.005244551	0.002499361
ENST00000568871	FAM214A	1.321785522	0.008245513	0.000714103
ENST00000568637	FAM214A	1.170026681	0.109389034	0.001432497
ENST00000566948	FAM214A	1.20862148	0.0342101	0.000999744
ENST00000333229	BRWD1	1.082051561	0.209134938	0.002499361
ENST00000377780	DIS3	1.186558259	0.010130467	0.001666241
ENST00000339526	GLUL	1.145108512	0.331809828	0.001697887
ENST00000480461	TRA2B	1.620475353	0.013192522	0.000454429
ENST00000546705	CORO1C	1.189891939	0.257191324	0.002100806
ENST00000422355	MORF4L2	1.127719629	0.424177507	0.002538336
ENST00000488331	MORF4L2	1.135438982	0.372520555	0.002229214
ENST00000543834	M6PR	1.262095196	0.039209743	0.000555414
ENST00000538253	PTPN22	1.186402283	0.036441681	0.004998722
ENST00000537196	IKBKAP	1.259405876	0.003665245	0.001666241
ENST00000552275	ATP2B1	1.149083448	0.205637585	0.001055143
ENST00000492227	TRIM33	1.152502844	0.099385164	0.00124968
ENST00000448530	VCP	-1.124322041	0.148841998	0.00083312
ENST00000358901	VCP	1.017316508	0.852793554	0.004500042
ENST00000589493	BECN1	1.08324841	0.430017318	0.003106066
ENST00000591085	BECN1	-1.184157905	0.161635234	0.00124968
ENST00000437070	RNF168	-1.410967143	0.011205428	0.002499361
ENST00000420432	PBX2	-1.261739635	0.008984627	0.000499872
ENST00000478700	MICU2	1.108074788	0.208559548	0.002499361
ENST00000469371	DLG1	1.011628309	0.824227507	0.004125604

Isoform ID	Gene Name	Fold Change	p-value (t test)	Two step significance threshold
ENST00000552414	SLC38A2	1.251099456	0.062766576	0.000555414
ENST00000415541	SIK3	-1.069147155	0.148589267	0.000801227
ENST00000446921	SIK3	-1.048271457	0.602665138	0.003249706
ENST00000485363	SIK3	-1.198447596	0.10409737	0.000561317
ENST00000424022	LBR	-1.053319448	0.68301778	0.004998722
ENST00000487054	LBR	-1.241935213	0.084745221	0.00124968
ENST00000485512	MTRF1L	-1.091302238	0.214157249	0.0034089
ENST00000314583	HCLS1	-1.015757572	0.884702212	0.004998722
ENST00000551209	GIT2	-1.025702321	0.793304194	0.004998722
ENST00000550186	GIT2	-1.148235012	0.19879424	0.001252631
ENST00000268957	TOB1	-1.375230138	0.08018759	0.002499361
ENST00000432873	SRSF7	1.345125574	0.014043988	0.000714103
ENST00000409276	SRSF7	-1.079673445	0.527209832	0.003842967
ENST00000415527	SRSF7	1.057444225	0.685765813	0.004998722
ENST00000447699	IARS	1.33048646	0.019335333	0.00124968
ENST00000459637	COX7A2	1.408842596	0.017809478	0.000999744
ENST00000587497	PTPN2	1.205783065	0.11868678	0.000999744
ENST00000320027	HSPH1	-1.001402241	0.990936623	0.004998722
ENST00000423591	FAM120AOS	1.305929145	0.012541181	0.00083312
ENST00000428378	FAM120AOS	-1.005630131	0.922514976	0.004998722
ENST00000556702	MAX	-1.18563622	0.185007626	0.00124968
ENST00000536607	TCP1	-1.242185046	0.216768937	0.001282348
ENST00000317610	RTN4	1.168175571	0.119713305	0.000999744
ENST00000511980	GAK	1.112378792	0.148579879	0.000753579
ENST00000576870	KANSL1	1.538799025	0.011088884	0.000434504
ENST00000432791	KANSL1	1.154146654	0.204311746	0.001068687
ENST00000376444	GRIPAP1	1.591658459	0.00060283	0.00083312
ENST00000555301	CHD8	1.204869315	0.046914713	0.00124968
ENST00000399982	CHD8	-1.112306605	0.216198508	0.002688137
ENST00000285681	USP25	1.220500278	0.001365026	0.00124968
ENST00000425608	RBM6	-1.256786994	0.066665455	0.00083312
ENST00000454079	RBM6	1.048168557	0.505096178	0.002853479
ENST00000464988	RBM5	1.056468439	0.698620611	0.003605741
ENST00000372764	PLAU	-1.165615202	0.274064815	0.004998722
ENST00000474835	USP16	1.270561181	0.018525276	0.00083312
ENST00000372724	KAT6B	1.089395401	0.300468947	0.002410158
ENST00000510080	PAIP2	-1.322879588	0.06048671	0.001057105
ENST00000464102	SACM1L	1.165212916	0.031865911	0.001666241
ENST00000587491	SEC14L1	-1.113408603	0.197798985	0.001666241
ENST00000479815	HMGN2	-1.496516746	0.004370489	0.000714103
ENST00000312046	OXR1	1.06382935	0.338284947	0.002499361
ENST00000589892	BIRC5	1.152917831	0.150393861	0.002499361
ENST00000470570	MTR	1.139339103	0.018917003	0.000999744
ENST00000327320	EED	1.197555947	0.002857148	0.00124968

Isoform ID	Gene Name	Fold Change	p-value (t test)	Two step significance threshold
ENST00000487938	VIM	1.778492664	0.000783823	0.000555414
ENST00000394601	PTBP1	1.099192689	0.567118645	0.003365386
ENST00000585856	PTBP1	-1.202666317	0.309312564	0.001835517
ENST00000338051	C5orf24	-1.10329574	0.101061971	0.002499361
ENST00000590485	SAFB	-1.09192626	0.499935764	0.002648274
ENST00000452673	CANX	1.001198945	0.991775752	0.004998722
ENST00000511663	SH3BP2	-1.199930083	0.118530375	0.000975654
ENST00000515183	SH3BP2	1.388006081	0.024416829	0.00062484
ENST00000557460	SRSF5	-1.479737314	0.083028836	0.000454429
ENST00000556587	SRSF5	-1.104552239	0.136360219	0.000739652
ENST00000295025	REL	1.030265763	0.787662992	0.004998722
ENST00000341360	ATP2B4	-1.069366825	0.524789676	0.004169021
ENST00000507716	TARS	1.110965089	0.200740128	0.001370799
ENST00000505012	TARS	-1.07052745	0.483679082	0.003302911
ENST00000558813	DUT	1.359415775	0.033112634	0.00124968
ENST00000531133	SERPING1	1.594808974	0.001058382	0.001327887
ENST00000534460	RCOR3	-1.24867628	0.168043096	0.001431158
ENST00000560473	KLF13	1.628406352	0.003127497	0.001666241
ENST00000562743	USP10	-1.195529558	0.109680662	0.000999744
ENST00000519618	CHRAC1	1.317557253	0.012988252	0.00124968
ENST00000220913	CHRAC1	-1.024329034	0.824679999	0.004998722
ENST00000562879	RPL13	1.086246511	0.596380319	0.003586618
ENST00000553956	CEP95	1.282377193	0.00655784	0.000454429
ENST00000579637	CEP95	1.242067922	0.108144783	0.000565858
ENST00000495112	TSN	-1.483802563	0.008636456	0.00083312
ENST00000546859	CCT2	1.167883046	0.108197205	0.000609414
ENST00000506523	RBM4	-1.058153602	0.483262727	0.002676016
ENST00000009180	CD9	1.512568811	0.033053519	0.002499361
ENST00000540056	MGST1	-1.341741659	0.057198629	0.000999744
ENST00000495673	YPEL5	1.292211629	0.072644149	0.00083312
ENST00000475721	EIF4G1	1.246763813	0.120199047	0.000796709
ENST00000459786	SP100	1.435848539	0.002651615	0.000555414
ENST00000397412	CRAMP1L	-1.307914305	0.000673341	0.00124968
ENST00000498626	CAPZA1	-1.326911421	0.042635026	0.000999744
ENST00000554589	KLHDC2	1.059795661	0.508170693	0.004927812
ENST00000359544	UBAP1	1.00277761	0.982892667	0.004998722
ENST00000487603	WSB1	-1.068789123	0.469274191	0.002495448
ENST00000469170	SRSF11	1.341332463	0.027550891	0.000333248
ENST00000475125	RARA	-1.604811588	0.00943542	0.00083312
ENST00000568704	CYLD	1.205221677	0.019839812	0.00062484
ENST00000563629	CYLD	1.045454353	0.694177251	0.004388971
ENST00000581383	SMCHD1	1.135691652	0.271863351	0.001666241
ENST00000583800	SMCHD1	1.056265647	0.614708437	0.003589619
ENST00000490422	ZMYM2	1.786494259	0.000225267	0.000714103

Isoform ID	Gene Name	Fold Change	p-value (t test)	Two step significance threshold
ENST00000497648	RABGGTB	-1.297309193	0.031273679	0.00062484
ENST00000505285	TCERG1	1.241293867	0.065117969	0.000714103
ENST00000540578	ANKRD12	-1.045454282	0.668753239	0.004998722
ENST00000359158	ANKRD12	1.215707255	0.019310916	0.00124968
ENST00000276520	TACC1	-1.062249991	0.210318526	0.001128256
ENST00000511305	KLHL2	1.068616529	0.500635028	0.004998722
ENST00000521338	PCM1	1.21388891	0.108550395	0.000620601
ENST00000541192	SENP6	1.282653563	0.006173136	0.00062484
ENST00000280377	USP15	1.25122929	0.033650101	0.000499872
ENST00000547908	SLC25A3	1.276594806	0.080395327	0.000714103
ENST00000483137	HNRNPA3	2.232194213	0.000230904	0.00124968
ENST00000447833	PDS5B	1.330234284	0.009419929	0.00124968
ENST00000432286	TNRC6A	1.482031809	0.000529831	0.00083312
ENST00000415365	BZW2	-1.401572082	0.009164918	0.000555414
ENST00000547394	CUX1	-1.247346126	0.004747558	0.00083312
ENST00000354719	MST4	1.155629991	0.112653414	0.002499361
ENST00000496850	MST4	-1.587517921	0.003121563	0.00124968
ENST00000554703	NAA30	-1.374659497	0.001348623	0.001666241
ENST00000354480	RBM3	-1.033228397	0.747846221	0.003754213
